# NIR‐Activatable, Sequence‐Specific Metal‐Nucleic Acid Scaffolds for Responsive Uncaging

**DOI:** 10.1002/anie.202514717

**Published:** 2025-09-16

**Authors:** Arpit Sharma, Man Kshetri, Deepak Karna, Md Al Amin, Shirin Akter, Hanbin Mao, Yao‐Rong Zheng

**Affiliations:** ^1^ Department of Chemistry and Biochemistry Kent State University Kent Ohio 44242 USA

**Keywords:** DNA‐mediated electron transfer, Molecular machines, Near infrared, Platinum(IV) complexes

## Abstract

Precise molecular activation with both analyte specificity and spatiotemporal control remains a major challenge in responsive diagnostics, targeted therapies, and the study of complex biological systems. Traditional photo‐uncaging strategies offer excellent temporal resolution but suffer from limited tissue penetration and poor biological specificity, while analyte‐responsive platforms provide molecular selectivity without external control. Here, we introduce sequence‐responsive diagnostic uncaging—a unique approach that integrates nucleic acid recognition with near‐infrared (NIR)‐triggered molecular activation within a metal‐nucleic acid scaffold. This platform is built upon a first‐of‐its‐kind Pt(IV)‐DNA molecular scaffold, modularly assembled via click chemistry, and integrates a Pt(IV)‐caged reporter, a nucleic acid recognition domain, and an NIR antenna (e.g., IR800). Notably, DNA‐mediated electron transfer (DNA‐MET) provides a long‐range ET pathway to direct photoreduction of the Pt(IV) centers, enabling “responsive uncaging” that occurs only upon hybridization with a fully complementary DNA or miRNA strand. Upon NIR irradiation, the duplexed nucleic acid system facilitates electron transfer from the excited antenna to Pt(IV), triggering the release of fluorescent reporters. Using two Pt(IV)‐caged fluorophores (MCA and BDP), we demonstrate efficient uncaging and high sequence specificity in both solution and live cells. This platform offers a powerful and versatile photochemical tool that seamlessly bridges diagnostics and molecular activation, with broad implications for precision medicine, targeted drug delivery, and next‐generation biosensing technologies.

## Graphical abstract

We demonstrate that long‐range nucleic acid‐mediated electron transfer can drive Pt(IV) photoreduction, establishing a new class of metal‐nucleic acid scaffolds capable of “responsive uncaging,” where hybridization with target DNA or miRNA directly triggers payload release under NIR irradiation.

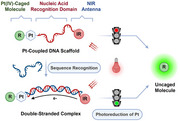

## Introduction

Precise control over when, where, and how molecular activation occurs is essential for advancing diagnostics, targeted therapies, and our understanding of complex biological processes.^[^ ^1^, ^2^, ^3^, ^4^ ^]^ Currently, controlled molecular activation methods often utilize photo‐uncaging strategies,^[^ ^4^, ^5^, ^6^, ^7^ ^]^ which provide high spatial and temporal resolution but are frequently limited in biological specificity and tissue penetration due to their dependence on UV or visible light.^[^ ^8^, ^9^, ^10^, ^11^ ^]^ Alternatively, analyte‐specific activation employs molecular triggers such as reactive oxygen species (ROS),^[^ ^12^, ^13^, ^14^, ^15^ ^]^ RNA sequences,^[^ ^1^, ^16^, ^17^, ^18^, ^19^ ^]^ proteins,^[^ ^20^, ^21^ ^]^ and bioorthogonal chemical reactions,^[^ ^22^, ^23^ ^]^ offering exceptional molecular selectivity. However, these methods often lack integrated spatiotemporal control. Emerging strategies aim to combine molecular‐specific recognition with controlled photoactivation, as demonstrated by recent approaches such as responsive visible‐light activatable photocages and light‐activated bioorthogonal chemistry, which integrate click chemistry with photochemical activation.^[^ ^24^, ^25^, ^26^ ^]^ Nevertheless, these innovative systems still require externally introduced chemical stimuli and activation via UV or visible light.^[^ ^27^, ^28^, ^29^, ^30^, ^31^, ^32^, ^33^, ^34^ ^]^ The development of methods that integrate endogenous analyte specificity with precise spatiotemporal activation through near‐infrared (NIR) irradiation remains unreported and represents a multivariable, logic‐gated actuation, potentially transforming molecular diagnostics, targeted therapies, and precision medicine.

Light‐responsive DNA scaffolds represent powerful tools for precise biological control,^[^ ^35^, ^36^, ^37^, ^38^, ^39^, ^40^ ^]^ leveraging their inherent sequence‐specific recognition capabilities to offer unrivaled potential in sensing,^[^ ^41^, ^42^, ^43^, ^44^ ^]^ targeted therapeutic delivery,^[^ ^45^ ^]^ and synthetic biology.^[^ ^46^, ^47^, ^48^, ^49^ ^]^ However, traditional DNA‐based molecular systems primarily utilize UV light activation,^[^ ^37^ ^]^ limiting their biological applications due to poor tissue penetration, cytotoxicity risks, and potential DNA damage.^[^ ^37^, ^50^ ^]^ Recently, NIR‐activated DNA systems have emerged as promising alternatives, providing enhanced biocompatibility, deeper tissue penetration, and improved safety profiles.^[^ ^4^, ^51^ ^]^ Nevertheless, current NIR‐triggered DNA nanoassemblies largely rely on indirect methods such as photon upconversion nanoparticles, which convert NIR photons into UV activation signals.^[^ ^41^, ^52^ ^]^ These indirect approaches face challenges including low activation efficiency, complex assembly requirements, susceptibility to photobleaching, and limited spatial and temporal precision. Therefore, the development of DNA‐integrated, directly NIR‐responsive functional groups remains an important unmet need. Successfully addressing this gap would enable precise, programmable, and biocompatible molecular control, significantly advancing applications in molecular diagnostics, targeted therapies, and precision medicine.

Among photoactivatable systems,^[^ ^34^, ^53^, ^54^, ^55^, ^56^ ^]^ platinum complexes—especially Pt(IV) derivatives—are highly attractive due to their controlled photochemical reduction from inert Pt(IV) states to biologically active Pt(II) forms, extensively explored in therapeutic contexts.^[^ ^26^, ^57^, ^58^, ^59^, ^60^, ^61^, ^62^ ^]^ Most current research on Pt(IV) complexes focuses on their role as prodrugs for controlled release of active Pt(II)‐based chemotherapeutic agents, such as cisplatin and oxaliplatin, upon exposure to visible/NIR irradiation.^[^ ^57^, ^63^, ^64^, ^65^, ^66^, ^67^ ^]^ Recent developments have shown that small‐molecule Pt(IV) complexes can be efficiently photoreduced using either single‐photon excitation at 650 nm or two‐photon excitation at 880 nm, and nanoparticle‐based platforms have expanded their applicability to NIR‐triggered drug delivery.^[^ ^68^, ^69^ ^]^ Despite these advances, exploiting Pt(IV) complexes beyond conventional chemotherapy remains relatively unexplored.^[^ ^70^ ^]^ Their intrinsic redox‐sensitive properties uniquely position them as pivotal components for integration into DNA‐mediated electron transfer (DNA‐MET) processes, potentially facilitating long‐range photoredox reactions for effective molecular activations.^[^ ^71^, ^72^, ^73^ ^]^ However, such Pt‐integrated DNA‐based photoredox systems have not yet been reported.

**Figure 1:**
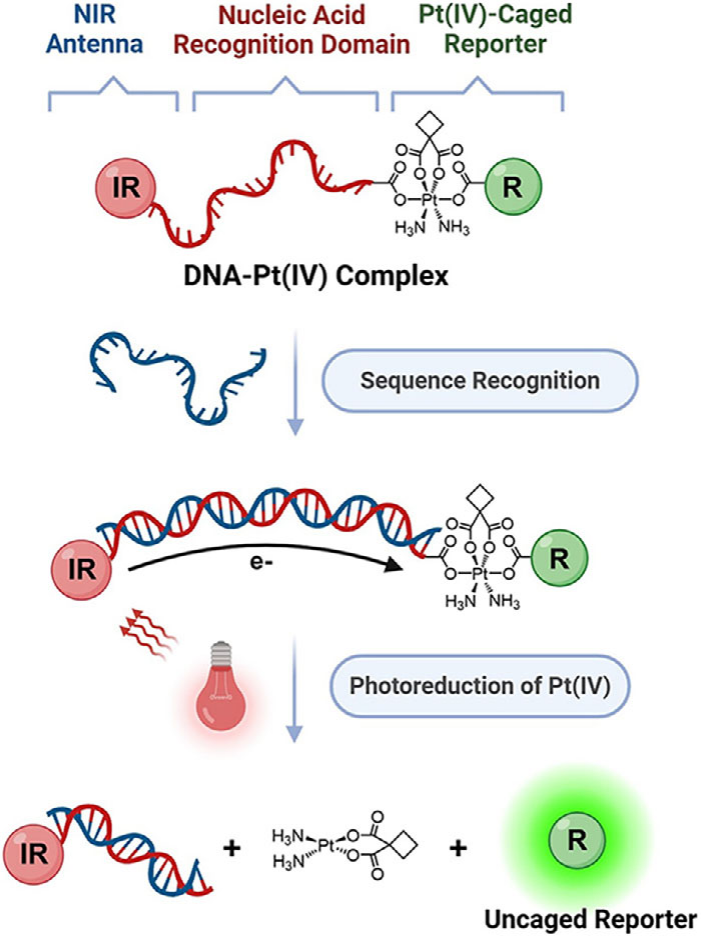
Schematic representation of the chemical design of the Pt(IV)‐coupled DNA scaffolds for sequence‐responsive diagnostic uncaging.

Herein, we introduce a novel chemical approach, called “sequence‐responsive diagnostic uncaging,” utilizing a pioneering Pt(IV)‐coupled DNA machine based on a dual‐module, DNA‐MET‐driven metal‐nucleic acid scaffold for DNA/RNA sequence recognition and NIR activation (Figure 1). This first‐of‐its‐kind scaffold comprises three key components: an NIR antenna (“IR”) attached to one terminus of a single‐stranded DNA (ssDNA), a Pt(IV)‐caged reporter (“R”) located at the opposite terminus, and a nucleic acid recognition domain. Sequence‐specific hybridization with a complementary nucleic acid strand (DNA or miRNA) induces the formation of a double‐stranded (ds) structure, enabling efficient DNA‐mediated electron transfer. Upon NIR irradiation, the antenna (“IR”) absorbs photons and gets excited to initiate photoinduced electron transfer (PET) through the duplex, leading to the reduction of the Pt(IV)‐caged reporter. This reduction event subsequently uncages the reporter (“R”), generating a fluorescence turn‐on signal indicative of successful nucleic acid recognition. By uniquely integrating analyte‐specific recognition with precise spatiotemporal NIR activation, this strategy effectively addresses the previously described limitations, significantly advancing nucleic acid‐controlled molecular diagnostics and targeted therapeutic applications.

## Results and Discussion

### Synthesis and Characterization of the Clickable Pt(IV)‐Caged Reporters

**Figure 2:**
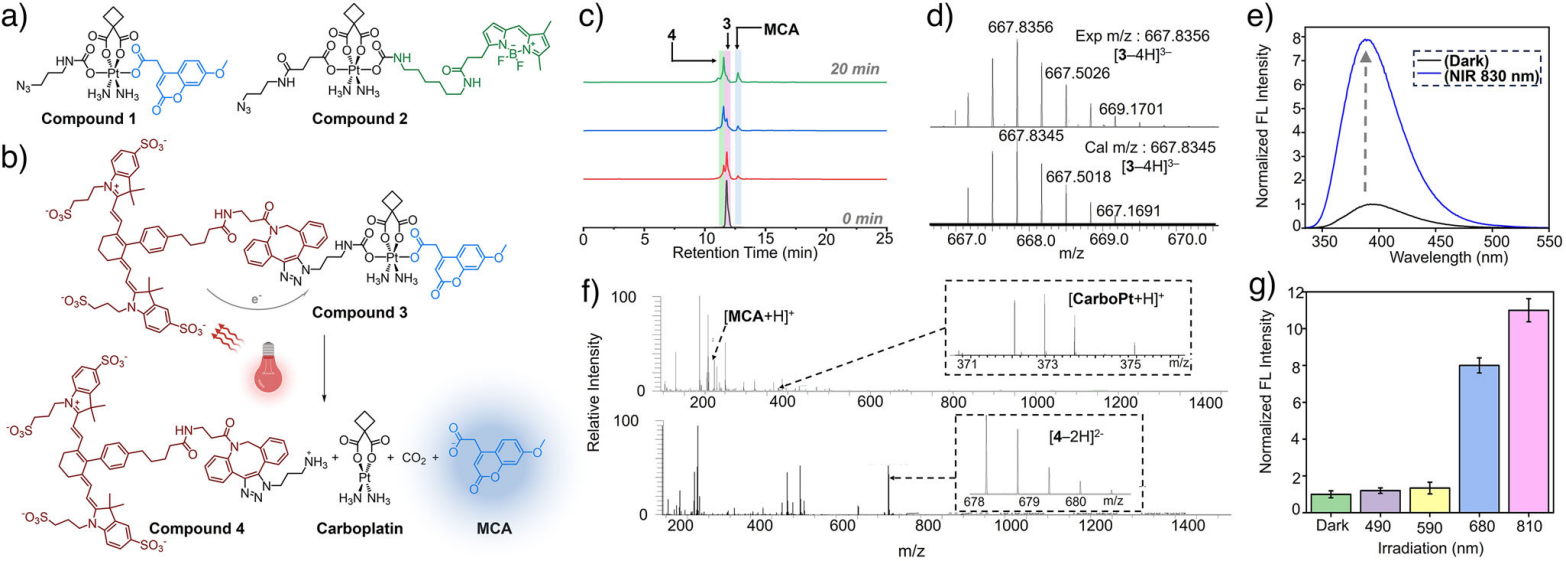
Clickable Pt(IV)‐caged reporters enabling NIR‐controlled molecular activation via NIR antenna‐mediated photoreduction. a) Chemical structures of clickable Pt(IV)‐caged reporters **1** and **2**, bearing MCA (carboxylate‐based) and BDP (amine‐based) respectively, with reversible axial carboxylate or carbamate linkages to the Pt(IV) core. b) NIR‐triggered photoreduction of Cy7‐clicked Pt(IV)‐caged MCA (**3**), releasing the carboxylate MCA reporter (see Figure 4a for the release of the amino BODIPY reporter). c) HPLC analysis of Compound **3** photoreduction under NIR irradiation (0, 5, 10, and 20 min). d) HR‐ESI‐MS spectra of Compound **3** (top, measured;, bottom, simulated). e) Fluorescence turn‐on from MCA released from **3** under NIR irradiation (830‐nm Irradiation, 15.3 mJ cm^−^^2^, 2 min). f) HR‐ESI‐MS spectra of photoreduction products in negative (*bottom*) or positive ion(*top*) modes. g) Wavelength‐selective fluorescence activation of Compound **3**. Data are presented as mean ± SD from *n* = 3 independent experiments.

Two distinct Pt(IV)‐caged reporters (Figure 2a) were synthesized for click‐assembly with DNA scaffolds: Compound **1** incorporates a carboxylate‐based coumarin reporter, 7‐methoxycoumarin‐4‐acetic acid (MCA), while Compound **2** features an amine‐based BODIPY fluorophore (BDP). Both compounds are equipped with azide handles for strain‐promoted azide–alkyne cycloaddition (SPAAC) click‐ligation to the DNA scaffold. These designs enable direct monitoring of Pt(IV) photoreduction via fluorescence turn‐on upon responsive uncaging. Notably, this pair showcases the versatility of Pt(IV) photocages through reversible axial carboxylate/carbamate connectivity, allowing release of either carboxylate (MCA) or amine‐based (BDP) cargos, as shown in Figure 2a. MCA was selected for its UV absorption profile, which facilitates precise wavelength‐selectivity studies across the visible‐to‐NIR range. In our recent work, we reported NIR‐triggered uncaging of glycine 7‐amido‐4‐methylcoumarin, an amine‐based coumarin reporter, from a Pt(IV)–Cy7 photocage.^[^ ^70^ ^]^ However, when adapted to DNA scaffolds, that amino analog, due to its positive charge, exhibited strong quenching upon binding to negatively charged duplex DNA. To overcome this, we reversed the axial carboxylate/carbamate connectivity at the Pt(IV) center and engineered to release the negatively charged carboxylate‐based MCA reporter, which shows minimal quenching in the presence of duplex DNA. In contrast, BDP serves as an excellent choice for in vitro imaging due to its superior photostability and emission in the biologically relevant range. Together, these two constructs exemplify the chemical and wavelength flexibility, as well as functional potential, of Pt(IV)‐coupled DNA scaffolds.

As outlined in Figure S1, two distinct synthetic routes were developed to prepare the clickable Pt(IV)‐caged reporters. Compound **1** was synthesized using a newly developed procedure that reverses the axial carboxylate/carbamate connectivity used in Compound **2**. To synthesize Compound **1**, carboxylic acid‐containing bioactive molecules were reacted with TBTU, DIPEA, and CarboPt(IV)‐Az‐O—a Pt(IV) complex bearing a hydroxyl ligand. This reaction efficiently forms carboxylate‐conjugated products under mild conditions (50 °C, open to air), yielding over 50%. The resulting Compound **1** was purified via flash column chromatography and fully characterized by multinuclear NMR (^1^H, ^13^C, and ^195^Pt) and electrospray ionization mass spectrometry (HR‐ESI‐MS) (Figure S4). In the ^1^H NMR spectrum, the characteristic shift of Pt‐bound ammine signals from δ = 6.0 to ∼6.5 ppm confirms carboxylate formation, while the ^1^⁹⁵Pt NMR signal at δ = 1979 ppm indicates retention of the Pt(IV) oxidation state. HR‐ESI‐MS further verified the composition through well‐defined isotopic patterns. For Compound **2**, amine‐containing bioactive molecules were mixed with MSC‐CarboPt(IV)‐azide, which carries a carbonate ligand. This reaction efficiently produces carbamate‐protected products under mild conditions. Compound **2** was similarly purified by flash chromatography and characterized by ^1^H, ^13^C, and ^195^Pt NMR, along with HR‐ESI‐MS. Together, these synthetic strategies offer facile and versatile methods for protecting (i.e., caging) carboxylate‐ or amine‐containing bioactive molecules using Pt(IV) complexes.

To evaluate the NIR‐uncaging capability of our Pt(IV)‐caged reporters, we performed a pilot study using Compound **1** click‐ligated with Cy7‐DBCO to generate Compound **3** (Figure S5). In this design, Cy7 acts as an NIR antenna (Figure 2b), enabling PET that reduces the Pt(IV) center and triggers release of the MCA fluorophore. We first characterized the deprotection process of compound **3** using HPLC and MS before and after NIR irradiation. HPLC analysis initially showed a single peak at 11.78 min corresponding to intact Compound **3** (Figure 2c,d). Upon irradiation, this peak gradually decreased, while two new peaks emerged at 11.56 min and 12.7 min, assigned to free MCA and Compound **4**, respectively. After 20 min of irradiation, only MCA and **4** remained, indicating complete uncaging. MS analysis further confirmed the photoreduction pathway. In negative ion mode, the signal for **3** disappeared, replaced by a peak corresponding to [**4**–2H]^2−^. In positive mode, clear signals for MCA and carboplatin were detected (Figures 2f and S6), validating the identities of the photoreduced products and supporting the reaction scheme shown in Figure 2b. In addition, fluorescence spectroscopy was used to monitor the photoreduction and release of MCA. Samples were analyzed before and after NIR irradiation using a fluorescence spectrophotometer, which revealed an ∼8‐fold increase in MCA fluorescence for Compound **3** (Figure 2e). To examine wavelength selectivity, we irradiated Compound **3** with LEDs spanning the visible to near‐infrared range. Fluorescence activation was maximal under NIR light, consistent with strong Cy7 absorption. No activation was observed under 490 or 590 nm light, where Cy7 absorption is minimal (Figure 2g). Furthermore, we conducted additional analyses to evaluate their dark stability against various biological reductants and in cell culture media. As shown in Figure S7, Compound **3** remained predominantly caged after 24 h of incubation with ascorbic acid, glutathione, or complete culture medium, retaining full fluorescence activation only upon NIR irradiation.

Overall, the ability to reverse axial carboxylate/carbamate connectivity provides a modular Pt(IV)‐based platform for selectively releasing either amine‐ or carboxylate‐containing cargos, enabling broad chemical adaptability. This versatile design retains high dark stability under physiological conditions while enabling efficient NIR‐triggered uncaging dependent on both the light‐harvesting antenna and Pt(IV) center. Together, these features make the system a powerful foundation for advancing the DNA–Pt scaffold developed in this work.

### Pt(IV)‐Coupled DNA Scaffolds Enabling Sequence‐Responsive Diagnostic Uncaging in Cell Extracts

**Figure 3:**
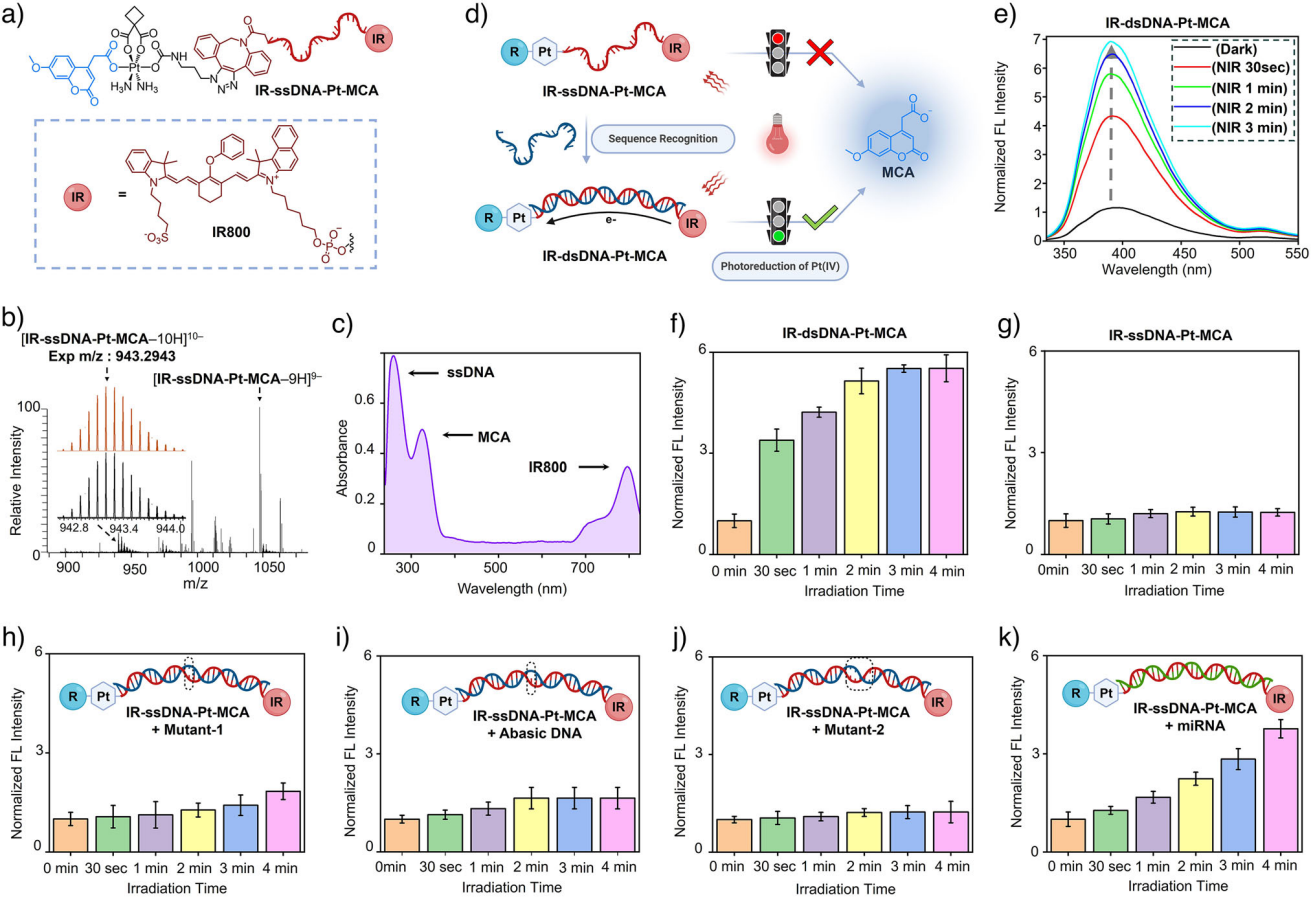
Pt(IV)‐clicked DNA scaffolds enabling sequence‐responsive diagnostic uncaging. a) Structure of the three‐component DNA scaffold (IR‐ssDNA‐Pt‐MCA), featuring an IR800 NIR antenna (IR), a clicked Pt(IV)‐caged MCA (Pt‐MCA), and a single‐stranded DNA linker (ssDNA). b) HR‐ESI‐MS spectra of IR‐ssDNA‐Pt‐MCA complex. c) UV–vis absorption spectrum of IR‐ssDNA‐Pt‐MCA in aqueous solution. d) Scheme illustrating duplex formation (IR‐dsDNA‐Pt‐MCA) via complementary DNA strand recognition, enabling NIR‐triggered MCA uncaging through DNA‐mediated electron transfer from IR to Pt; no uncaging occurs without complementary strand. e) Fluorescence spectra showing time‐dependent MCA release from IR‐dsDNA‐Pt‐MCA under NIR irradiation (830‐nm, 15.3 mJ cm^−^^2^, 0–3 min). f) Quantitative analysis of MCA uncaging kinetics from IR‐dsDNA‐Pt‐MCA under NIR irradiation. g) No MCA release from IR‐ssDNA‐Pt‐MCA without complementary strand under NIR irradiation. h) Slight MCA uncaging from IR‐ssDNA‐Pt‐MCA with mutant‐1 strand (with one base‐pair mismatch, dotted circle) under NIR irradiation. i) Little MCA uncaging from IR‐ssDNA‐Pt‐MCA containing an abasic nucleotide (dotted circle) under NIR irradiation. j) No MCA uncaging from IR‐ssDNA‐Pt‐MCA with mutant‐2 strand (with 3 base‐pair mismatches in dotted circle) under NIR irradiation. k) MCA uncaging from IR‐ssDNA‐Pt‐MCA in the presence of an miRNA21 strand under NIR irradiation. Please see Table S1 for detailed sequences. Data are presented as mean ± SD from *n* = 3 independent experiments.

Building on this chemical versatility, we next harnessed the modular, clickable Pt(IV)‐caged reporters to construct the first Pt(IV)‐coupled DNA scaffolds for proof‐of‐concept sequence‐responsive diagnostic uncaging. As illustrated in Figure 3a, this novel construct consists of three key components: an NIR dye (IR800) conjugated to the 5′ end of a single‐stranded DNA (ssDNA), a Pt(IV)‐caged MCA reporter click‐ligated to the 3′ end, and a central DNA sequence designed to recognize a specific complementary nucleic acid analyte. IR800, shown in Figure 3a, is a Cy7‐derived NIR dye selected for this study due to its widespread use in DNA conjugation and commercial availability for direct oligonucleotide modification. In addition, we chose a sequence complementary to miRNA21, a 22‐nt long biologically relevant microRNA implicated in various cancers.^[^ ^49^, ^74^, ^75^ ^]^ Upon hybridization with miRNA21 or its complementary DNA, the scaffold forms a double‐stranded structure that functions as a conductive “molecular wire.” This enables DNA‐MET from the IR800 antenna to the Pt(IV) center upon NIR irradiation, triggering reporter uncaging.

The synthesis of the Pt(IV)‐coupled DNA scaffold (IR‐ssDNA‐Pt‐MCA) followed a stepwise click‐ligation strategy (Figure S8). First, IR‐ssDNA with a terminal disulfide (IR‐ssDNA‐S‐S) was treated with TCEP·HCl to generate a free thiol, which was then conjugated with Maleimide‐PEG_4_‐DBCO to form IR‐ssDNA‐DBCO. After purification with Amicon 3 K filters to remove excess linker, the construct was reacted with excess of Compound **1**, an azide‐functionalized Pt(IV)‐caged MCA reporter, via SPAAC. The final product, IR‐ssDNA‐Pt‐MCA, was purified using a NAP‐5 column to remove unreacted reagents. Conjugation was confirmed by 18% denaturing PAGE gel, where a clear band shift was observed in IR‐ssDNA‐Pt‐MCA compared to IR‐ssDNA‐S‐S (Figure S8). Further confirmation was provided by HR‐ESI‐MS analysis (Figure 3b), which showed mass signals consistent with the expected conjugate. Characteristic absorption peaks for the DNA strand, MCA reporter, and IR800 antenna were observed, as shown in Figure 3c.

To test whether duplex formation is necessary for DNA‐MET, we conducted a series of fluorescence experiments (Figure 3d–k). Upon 830‐nm irradiation, the fully hybridized duplex (IR‐dsDNA‐Pt‐MCA) showed a 6‐fold increase in coumarin fluorescence within 4 min (Figure 3e), indicating efficient PET. In contrast, the single‐stranded construct (IR‐ssDNA‐Pt‐MCA) exhibited no significant fluorescence change (Figure 3g), confirming that duplex formation is essential for PET.

We next evaluated the sequence specificity of this metal‐nucleic acid platform using DNA variants with a single mismatch, three mismatches, or an abasic site. A single‐nucleotide mismatch reduced PET efficiency to a 1.8‐fold fluorescence increase (Figure 3h), while the abasic variant yielded a 1.65‐fold enhancement (Figure 3i). The three‐mismatch variant nearly abolished coumarin release (Figure 3j), showing fluorescence comparable to the single‐stranded control. These results underscore the essential role of duplex formation in facilitating Pt(IV) photoreduction by the excited NIR antenna and reveal the system's high sensitivity to base‐pairing fidelity, with even minor mismatches markedly reducing photoreduction efficiency.

As a proof‐of‐concept for its application potential, we next applied the platform to detect miRNA21—a biologically relevant molecular target. Upon hybridization with IR‐ssDNA‐Pt‐MCA and subsequent NIR irradiation, we observed a 4‐fold fluorescence increase (Figure 3k)—comparable to the fully matched DNA duplex—demonstrating that miRNA21 hybridization is sufficient to initiate uncaging. Next, we extended our investigation to complex biological environments by evaluating sequence‐responsive diagnostic uncaging using endogenous miRNA extracted from HeLa cells, known for their characteristic miRNA expression profiles.^[^ ^49^, ^74^, ^75^ ^]^ Following isolation with a commercial miRNA extraction kit (see Supporting Information for details), the HeLa‐derived miRNA was annealed with IR‐dsDNA‐Pt‐MCA at various molar ratios (1:1, 1.5:1, and 2:1, miRNA/DNA). At the 2:1 ratio, NIR irradiation triggered MCA release comparable to that achieved with synthetic miRNA controls (Figure S10). In contrast, lower ratios (1:1 and 1.5:1) resulted in reduced fluorescence signals, indicating that optimal stoichiometry is critical for efficient activation with cellular RNA. These results confirm that the system can selectively sense endogenous miRNA21.

To sum up, we established a first‐of‐its‐kind metal–nucleic acid scaffold using clickable Pt(IV)‐caged reporters. With the new scaffold, we demonstrated that high‐specificity duplex formation is essential for DNA‐MET, enabling the excited NIR dye to photoreduce the Pt(IV) center and trigger linker cleavage for cargo release. This new chemistry holds broad implications for chemical biology, including DNA technology, molecular sensing, and drug delivery. In this work, we demonstrate its potential as a platform for miRNA‐responsive applications.

### Sequence‐Specific Recognition of miRNA Enables NIR‐Controlled Activation In Vitro

**Figure 4:**
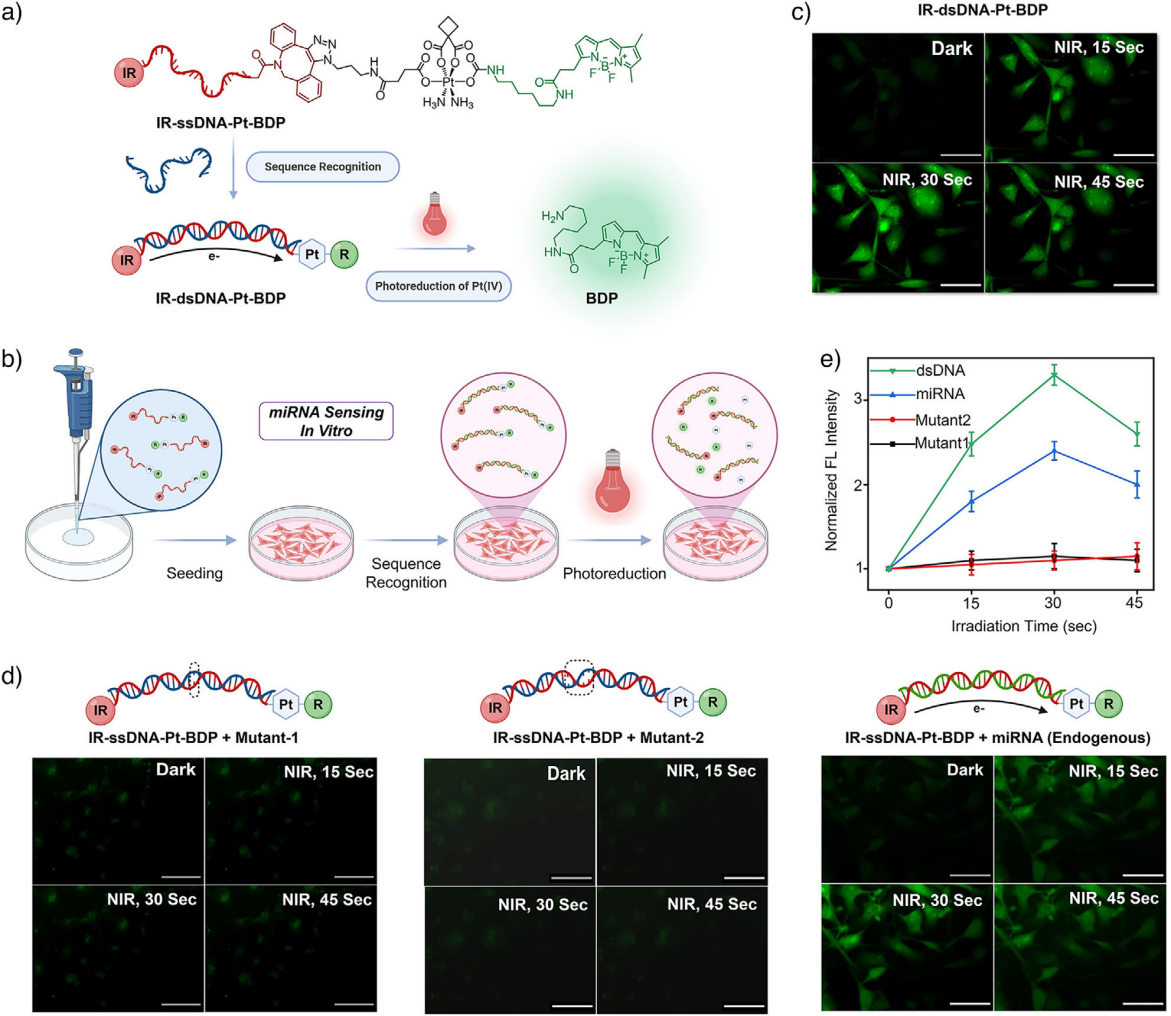
Cell‐based studies of responsive diagnostic uncaging in vitro: a) Schematic illustration of the three‐component DNA scaffold (IR‐ssDNA‐Pt‐BDP), comprising an IR800 NIR antenna (IR), a Pt(IV)‐caged fluorophore (BDP), and a single‐stranded DNA (ssDNA) linker. Upon hybridization with complementary DNA or miRNA21, the scaffold forms a duplex (IR‐dsDNA‐Pt‐BDP), enabling NIR‐triggered BDP uncaging via DNA‐mediated electron transfer from the antenna (IR) to Pt(IV). b) Schematic representation highlighting miRNA21 sequence‐specific recognition in cells by IR‐ssDNA‐Pt‐BDP, triggering BDP uncaging upon NIR irradiation. c) Fluorescence microscopy images demonstrating time‐dependent BDP release in vitro from IR‐dsDNA‐Pt‐BDP upon NIR irradiation (830‐nm LED, 15.3 mJ cm^−^^2^). d) Fluorescence imaging of BDP uncaging from IR‐ssDNA‐Pt‐BDP incubated with HeLa cells under NIR irradiation, showing responsive uncaging only upon recognition of target sequences. Controls with mismatched sequences (Mutant‐1, Mutant‐2) exhibit no significant BDP uncaging. e) Quantitative analysis of fluorescence intensity increase resulting from sequence‐specific BDP uncaging. Scale bars in (c) and (d) depict 100 µm. Data are presented as mean ± SD from *n* = 3 independent experiments.

Having validated the robust performance of Pt(IV)‐coupled DNA scaffolds in both solution and cell extracts, we next evaluated sequence‐responsive diagnostic uncaging in vitro. For this purpose, we employed Compound **2**, a Pt(IV) complex bearing an amine‐based BODIPY (BDP) reporter, chosen for its superior performance in fluorescence imaging. We first evaluated the stability of Compound **2** under cell‐free and intracellular conditions, in parallel with the free BDP reporter as a control. In cell‐free assays, Compound **2** remained stable for 24 h in PBS (with and without biological reductants), cell culture medium, and cell lysate, as determined by fluorescence measurements (Figure S11). Consistent with these findings, live‐cell imaging confirmed its high intracellular stability: while free BDP localized primarily to mitochondria, Compound **2** showed minimal colocalization even after 24 h (Figure S12), indicating that BDP was not prematurely released. With this stability established, Compound **2** was click‐conjugated to a DNA scaffold to generate IR‐ssDNA‐Pt‐BDP for the in vitro studies (Figure 4a). The final product was characterized by 18% denaturing PAGE gel (Figure S13), showing a clear band shift relative to IR‐ssDNA‐DBCO and IR‐ssDNA‐S‐S, confirming successful conjugation. Further confirmation was provided by ESI‐HR‐MS analysis (Figure S13), which showed mass signals consistent with the expected conjugate. Fluorescence studies revealed a 5‐fold increase in BDP emission within 30 sec of 830‐nm irradiation of the fully hybridized duplex (IR‐dsDNA‐Pt‐BDP) (Figure S14), consistent with efficient PET. Next, we assessed whether the newly developed DNA–Pt scaffold could reliably report BDP release after incubation in cell culture medium for varying durations. Samples were pre‐incubated for up to 24 h before NIR irradiation, and, as shown in Figure S15, the scaffold consistently produced a strong fluorescence turn‐on at all time points. To further confirm BDP release, a PAGE gel‐based assay was performed (Figure S16). Samples with and without NIR irradiation were separated by PAGE and imaged using either the BDP (Alexa Fluor 488) or NIR (650 nm) channels. In the NIR channel, DNA constructs remained visible both before and after irradiation. However, in the BDP channel, fluorescence from the construct was detectable only before irradiation but disappeared afterward, indicating the release of BDP and validating the NIR‐triggered uncaging mechanism.

We next assessed the construct's functionality in vitro. HeLa cells were treated with IR‐ssDNA‐Pt‐BDP under four conditions: i) hybridized with fully complementary DNA, ii) with single‐nucleotide mismatched DNA, iii) with triple‐nucleotide mismatched DNA, and iv) without any DNA hybridization. Briefly, as shown in Figure 4b, the DNA–Pt construct was loaded onto imaging dishes. After pre‐incubation, HeLa cells were seeded and incubated with these constructs at 37 °C with 5% CO_2_, followed by 830‐nm NIR irradiation. As shown in Figure 4c–e, cells treated with the fully matched construct exhibited a 3.5‐fold increase in BDP fluorescence, confirming successful uncaging in the cellular environment. In contrast, constructs hybridized with single‐ or triple‐mismatched strands showed no significant fluorescence change (Figure 4e), indicating high sequence specificity. Interestingly, even cells treated with IR‐ssDNA‐Pt‐BDP alone—without any added DNA—showed a 2.5‐fold fluorescence increase upon NIR exposure. This suggests that endogenous miRNA21 of HeLa cells effectively hybridized with the construct, initiating PET and triggering BDP uncaging.

Collectively, these results demonstrate that the Pt(IV)‐DNA scaffold enables reliable and selective photo‐uncaging of fluorescent reporters in biological systems. Moreover, the ability of endogenous miRNA to activate the system highlights its potential for miRNA‐responsive diagnostics and therapeutic applications, offering a powerful platform for sequence‐specific, light‐controlled molecular activation in vitro.

## Conclusion

We present a novel chemical strategy termed “sequence‐responsive diagnostic uncaging,” which enables precise molecular activation with both analyte specificity and spatiotemporal control via light. Central to this approach is a newly designed metal‐nucleic acid scaffold based on Pt(IV)‐ligated DNA strands. We explore photoactivatable Pt(IV) complexes as an unique light‐controlled chemical tool, distinct from the conventional focus on the therapeutic applications of Pt drugs. In this work, we developed clickable Pt(IV)‐caged reporters capable of masking and releasing carboxylate‐ or amine‐containing bioactive molecules while preserving an azide handle for SPAAC click reactions. The Pt(IV) complexes exhibit excellent stability in the dark under physiological conditions. Conjugation of these reporters to an ssDNA bearing a DBCO group on one end and an NIR antenna on the other yields a first‐of‐its‐kind modular Pt(IV)‐DNA molecular device. Upon hybridization with a complementary nucleic acid strand (DNA or miRNA), the system forms a duplex structure that facilitates DNA‐MET. NIR irradiation excites the antenna (IR800), initiating PET through the duplex, which reduces the Pt(IV) center and triggers uncaging of the reporter. This event results in a fluorescence turn‐on signal, confirming successful nucleic acid recognition.

To validate this platform, we synthesized two Pt(IV)–DNA scaffolds incorporating fluorescent reporters—MCA (carboxylate‐based) and BDP (amine‐based)—via reversible axial carboxylate/carbamate linkages to the Pt core and evaluated their uncaging efficiency in solution and in cell culture assays. Additionally, sequence specificity was evaluated using a panel of nucleic acid targets, including fully complementary DNA, miRNA21, single‐ and triple‐nucleotide mismatched strands, and abasic variants. Robust fluorescence turn‐on was observed only in the presence of fully complementary DNA or miRNA21, confirming successful sequence‐guided uncaging. In contrast, mismatched or abasic sequences failed to trigger reporter release, underscoring the high selectivity of this DNA‐mediated responsive uncaging system. Owing to this high specificity, the Pt–DNA scaffolds reliably detect miRNA21 from both cell extracts and in vitro, showcasing their potential as a platform for diagnostics and therapeutic applications.

Overall, this study presents the first NIR‐activated DNA scaffold chemically integrated with photoactive Pt(IV) complexes for sequence‐responsive diagnostic uncaging. This responsive uncaging approach establishes a new paradigm that unites molecular recognition with NIR‐triggered activation, bridging diagnostics and therapy within a single molecular platform. Unlike conventional strategies that offer either spatiotemporal control (e.g., photo‐uncaging) or molecular specificity (e.g., analyte‐triggered activation), this system achieves both. To enable this capability, the Pt–DNA scaffold incorporates several innovations. To our knowledge, this is the first demonstration of DNA‐MET being harnessed to drive Pt(IV) photoreduction, enabling nucleic acid‐guided activation with high sequence fidelity. The system exhibits mismatch discrimination down to the single‐nucleotide level and responds selectively to miRNA21 in different settings—including synthetic oligonucleotides, cell extracts, and endogenous targets in vitro. Reported nucleic acid‐based activation strategies, such as nanoflares and molecular beacons, typically rely on analyte‐triggered activation alone, producing an immediate signal upon hybridization without the ability to control when or where activation occurs. Notably, our DNA–Pt scaffold decouples recognition from activation: target hybridization primes the system, but NIR irradiation is required to initiate electron transfer and payload release. This dual‐gating mechanism provides additional spatiotemporal control, enabling on‐demand precision activation. The long‐range DNA‐MET offers ample opportunities to modulate responsive uncaging since the ET along the nanometer‐length pathway can be readily altered by factors such as DNA secondary structures, DNA‐ligand interactions, and solvent conditions. In addition, the scaffold features a modular, click‐compatible design that enables NIR‐triggered cleavage of both carboxylate and amine linkages, allowing broad payload compatibility. In sum, by coupling sequence‐specific hybridization with noninvasive photochemical control, this system offers a versatile and generic framework for programmable molecular activation—advancing the frontiers of precision diagnostics, targeted therapeutics, and DNA‐based molecular technologies.

## Supporting Information

Experimental procedures and characterization data include ^1^H, ^13^C, and ^195^Pt NMR spectra, HR‐ESI‐MS spectra, HPLC analysis, fluorescence spectroscopy, gel electrophoresis, and fluorescence imaging.

## Conflict of Interests

The authors declare no conflict of interest.

## Supporting information



Supporting Information

## Data Availability

The data that support the findings of this study are available in the supplementary material of this article.
