# Supplementary material for: NIR‐Activatable, Sequence‐Specific Metal‐Nucleic Acid Scaffolds for Responsive Uncaging
Source: Angew Chem Int Ed Engl. 2025 Sep 16;64(45):e202514717. doi: 10.1002/anie.202514717 (PMC12582009; doi:10.1002/anie.202514717)
Supplement: Supplementary file 1 — Supporting Information [file ANIE-64-e202514717-s001.docx]

**NIR-Activatable, Sequence-Specific Metal-Nucleic Acid Scaffolds for Responsive Uncaging**

Arpit Sharma, Man Kshetri, Deepak Karna, Md Al Amin, Shirin Akter, Hanbin Mao* and Yao–Rong Zheng*

Department of Chemistry and Biochemistry, Kent State University, Kent, Ohio 44242, United States

**Table of Contents**

[Fig S1.](#_Toc189667871) [[Synthetic route for preparing clickable Pt(IV) Compounds 1 & 2 3](#_Toc189667871)](#_Toc189667868)

[Fig S2.](#_Toc189667871)[[Characterization of CarboPt(IV)–MSC 4](#_Toc189667871)](#_Toc189667869)

[Fig S3.](#_Toc189667871)[[Characterization of CarboPt(IV)–Az–O 5](#_Toc189667871)](#_Toc189667870)

[Fig S4.[Characterization of Compound 1 6](#_Toc189667871)](#_Toc189667871)

[Fig S5.](#_Toc189667871)[[Characterization of Compound 3 7](#_Toc189667871)](#_Toc189667870)

[Fig S6. [Photoreduction of Compound 3 8](#_Toc189667871)](#_Toc189667871)

[Fig S7. [Fluorescence-based stability analysis of Compound 3 against biological reductants and in cell culture medium 9](#_Toc189667871)](#_Toc189667871)

[Fig S8. Synthesis and Characterization of IR–ssDNA–](#_Toc189667871)[Pt–MCA 10 Fig S9. [Fluorescence studies of IR–ssDNA-Pt-MCA with different DNA variants 11](#_Toc189667871)](#_Toc189667871)

[[Fig S10. miRNA extraction assay 11](#_Toc189667871)](#_Toc189667870)

[Fig S11. Fluorescence-based stability assessment of Compound 2 (5 µM) in the dark across various environments  [12](#_Toc189667871)](#_Toc189667871)

[Fig S12. Imaging-based stability assessment of Compound 2 in the dark](#_Toc189667871) *[in vitro](#_Toc189667871)* [through time-dependent mitochondrial colocalization analysis  [13](#_Toc189667871)](#_Toc189667871)

[Fig S13. Synthesis and characterization of IR–ssDNA-Pt-BDP  [14](#_Toc189667871)](#_Toc189667871)

[Fig S14. Fluorescence studies of IR–dsDNA–Pt–BDP before and after the irradiation  [15](#_Toc189667871)](#_Toc189667871)

[Fig S15. Fluorescence-based assessment of IR–dsDNA–Pt–BDP stability and NIR-triggered functionality in cell culture medium over time  [15](#_Toc189667871)](#_Toc189667871)

[Fig S16. Photoreduction of IR–dsDNA–Pt–BDP using PAGE gel–based assay  [16](#_Toc189667871)](#_Toc189667871)

[Table S1.](#_Toc189667871) [[DNA sequences 16](#_Toc189667871)](#_Toc189667868)

[[General information 17](#_Toc189667871)](#_Toc189667868)

[[Synthesis of CarboPt(IV)–(OH)](#_Toc189667871)_[2](#_Toc189667871)_ [17](#_Toc189667871)](#_Toc189667869)

[[Synthesis of CarboPt(IV)–MSC 17](#_Toc189667871)](#_Toc189667869)

[[Synthesis of CarboPt(IV)–Az–O 17](#_Toc189667871)](#_Toc189667870)

[[Synthesis of Compound 1 18](#_Toc189667871)](#_Toc189667871)

[[Synthesis of CarboPt(IV)–COOH 18](#_Toc189667871)](#_Toc189667870)

[[Synthesis of CarboPt(IV)–Azide 18](#_Toc189667871)](#_Toc189667871)

[[Synthesis of MSC–CarboPt(IV)–Azide 18](#_Toc189667871)](#_Toc189667871)

[[Synthesis of Compound 2 19](#_Toc189667871)](#_Toc189667871)

[[Synthesis of IR–ssDNA-Pt-MCA 19](#_Toc189667871)](#_Toc189667870)

[[Synthesis of IR–ssDNA-Pt-BDP 19](#_Toc189667871)](#_Toc189667871)

[Click-Assembly of Photoactivatable Pt(IV) Compound (3)………………………………….19 [Fluorescence studies of Compound 3 before and after NIR irradiation 20](#_Toc189667871)](#_Toc189667871)

[[Fluorescence Studies of Compound 3 in Response to LED Irradiation at Variable Wavelengths 20](#_Toc189667871)](#_Toc189667871)

[[HPLC and ESI–MS analysis of Photo–Uncaging of Compound 3 20](#_Toc189667871)](#_Toc189667871)

[Fluorescence-based stability assessment of Compound 3 in the dark 20](#_Toc189667871) [[Fluorescence studies of DNA–Pt–Scaffolds before and after NIR irradiation 20](#_Toc189667871)](#_Toc189667868)

[[Cell Culture 20](#_Toc189667871)](#_Toc189667868)

[[miRNA extraction assay 20](#_Toc189667871)](#_Toc189667868)

[[Fluorescence studies of IR–ssDNA–Pt–BDP + miRNA (from cells) 21](#_Toc189667871)](#_Toc189667868)

[[Photoreduction of IR–dsDNA–Pt–BDP using PAGE gel–based assay………………………21](#_Toc189667871)](#_Toc189667868) [Fluorescence-based stability assessment of Compound 2 in the dark 21](#_Toc189667871)  [[Cell imaging for assessing stability of Compound 2 in the dark in vitro 21](#_Toc189667868)](#_Toc189667868)

[[Fluorescence-based assessment of IR–dsDNA–Pt–BDP stability and NIR-triggered functionality in cell culture medium 22](#_Toc189667868)](#_Toc189667868)

[Cell imaging for validating functionality of the DNA-Pt scaffold](#_Toc189667868) *[in vitro](#_Toc189667868)* [22](#_Toc189667868)

Fig S1. Synthetic route for preparing the clickable Pt(IV) compounds (1 and 2), bearing MCA (carboxylate-based) and BDP (amine-based), with reversible axial carboxylate or carbamate linkages to the Pt(IV) core.


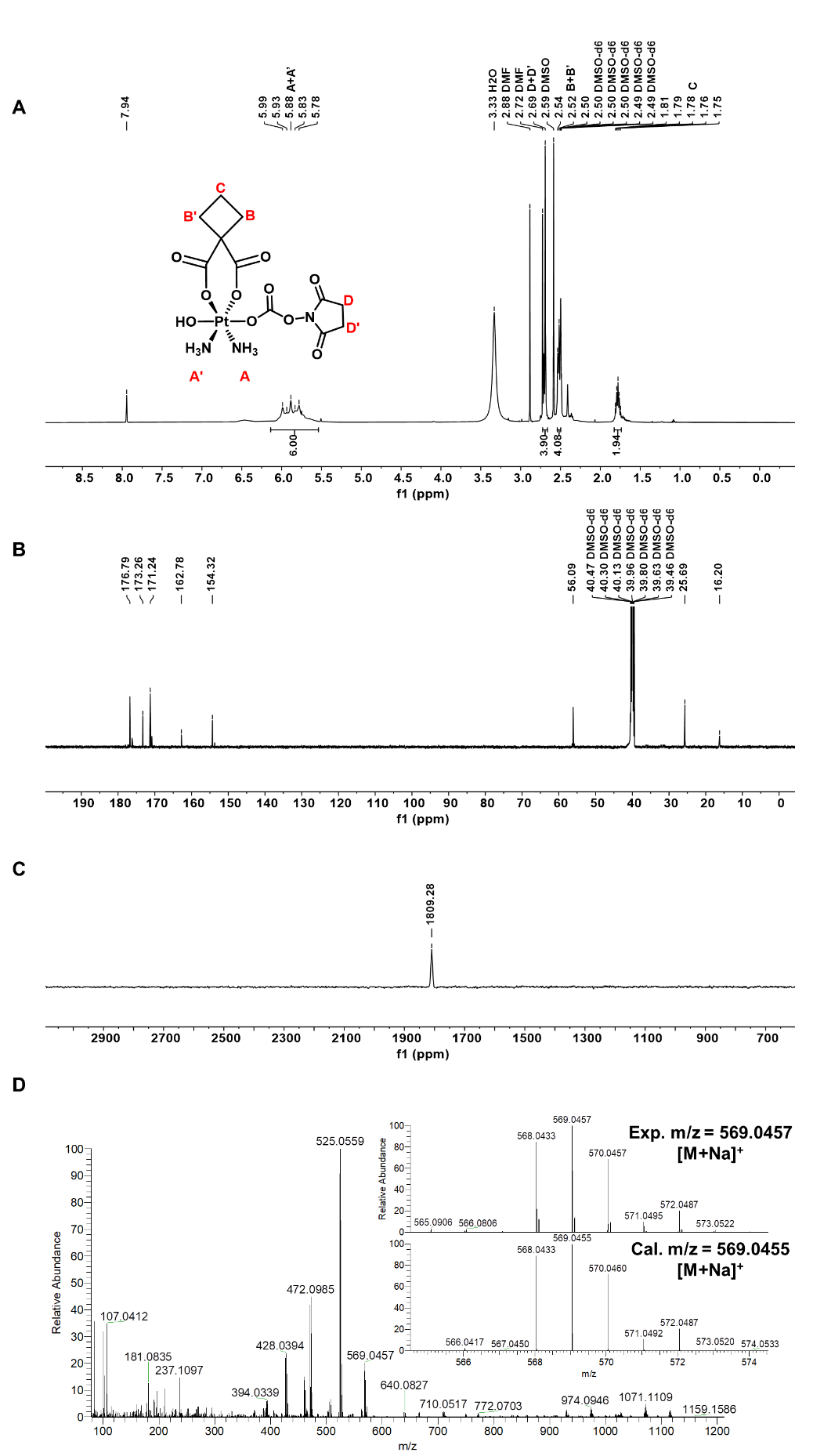


**Fig S2**. Characterization of CarboPt(IV)–MSC **A**. ^1^H NMR spectrum in DMSO–d_6_; **B.** ^13^C NMR spectrum in DMSO–d_6_; **C.** ^195^Pt NMR spectrum in DMSO–d_6_; **D.** High resolution ESI–MS spectra.


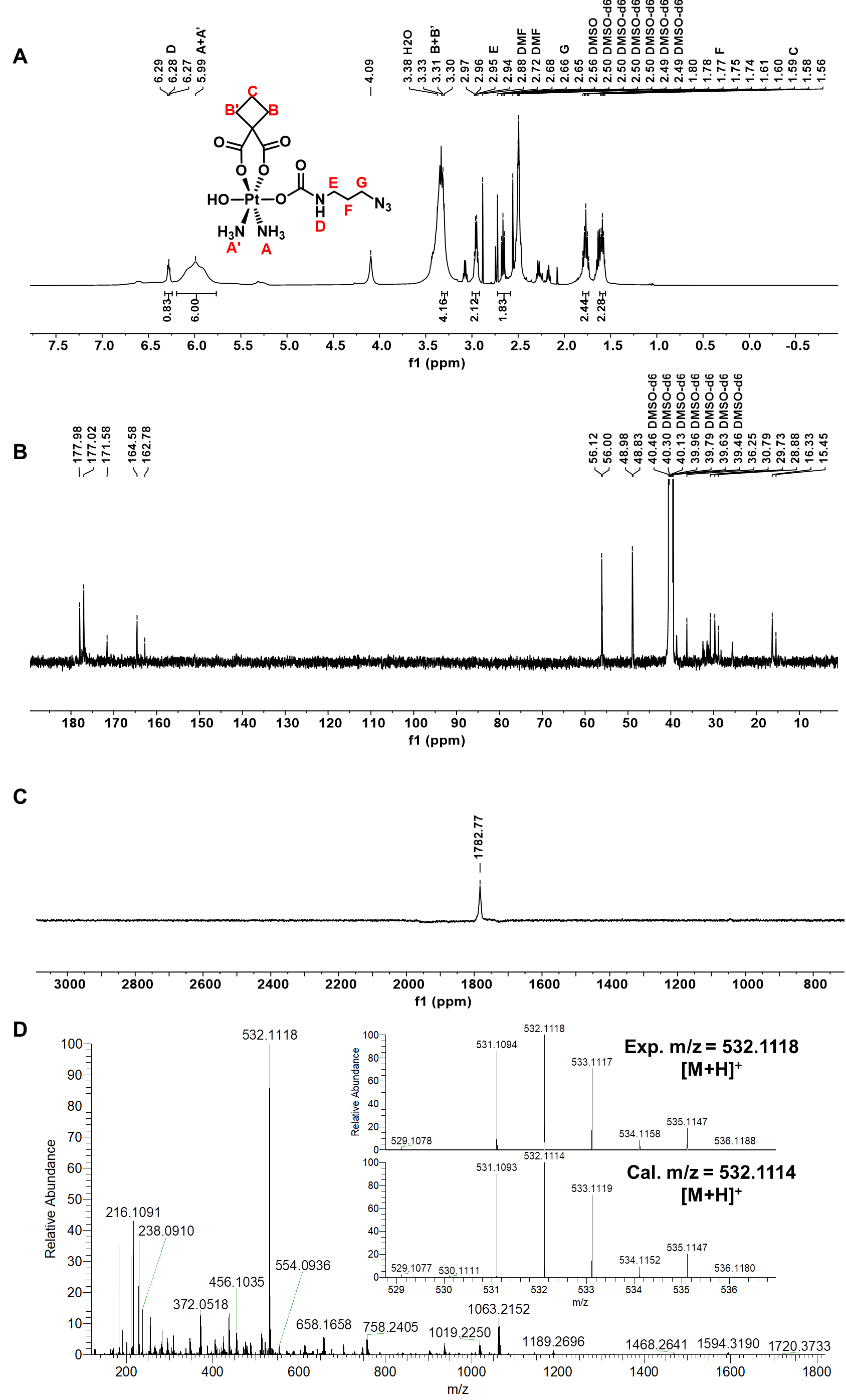


**Fig S3**. Characterization of CarboPt(IV)–Az–O: **A.** ^1^H NMR spectrum in DMSO–d_6_; **B.** ^13^C NMR spectrum in DMSO–d_6_; **C.** ^195^Pt NMR spectrum in DMSO–d_6_; **D.** High resolution ESI–MS spectra.


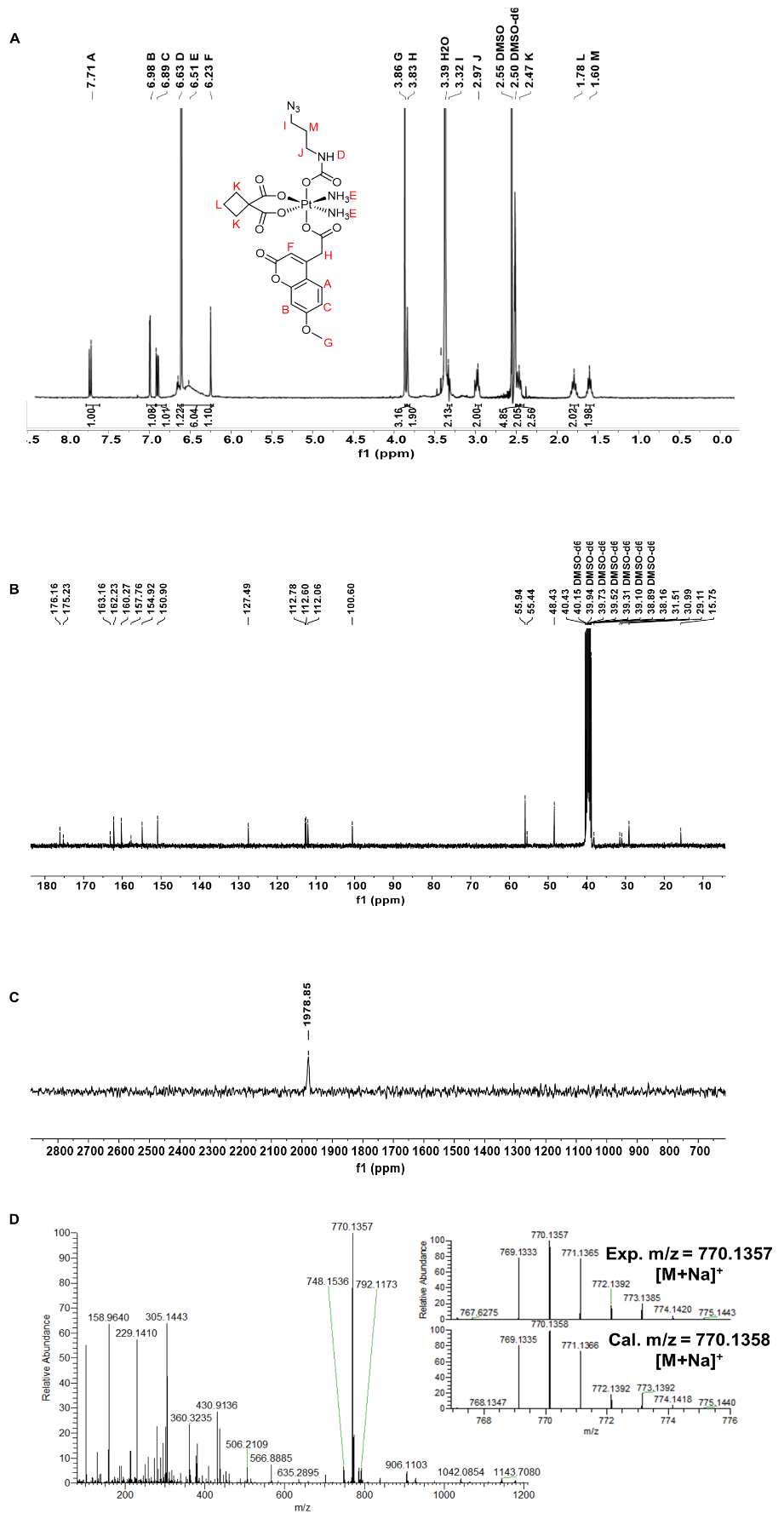


**Fig S4.** Characterization of Compound 1: **A.** ^1^H NMR spectrum in DMSO–d_6_; **B.** ^13^C NMR spectrum in DMSO–d_6_; **C.** ^195^Pt NMR spectrum in DMSO–d_6_; **D.** High resolution ESI–MS spectra.


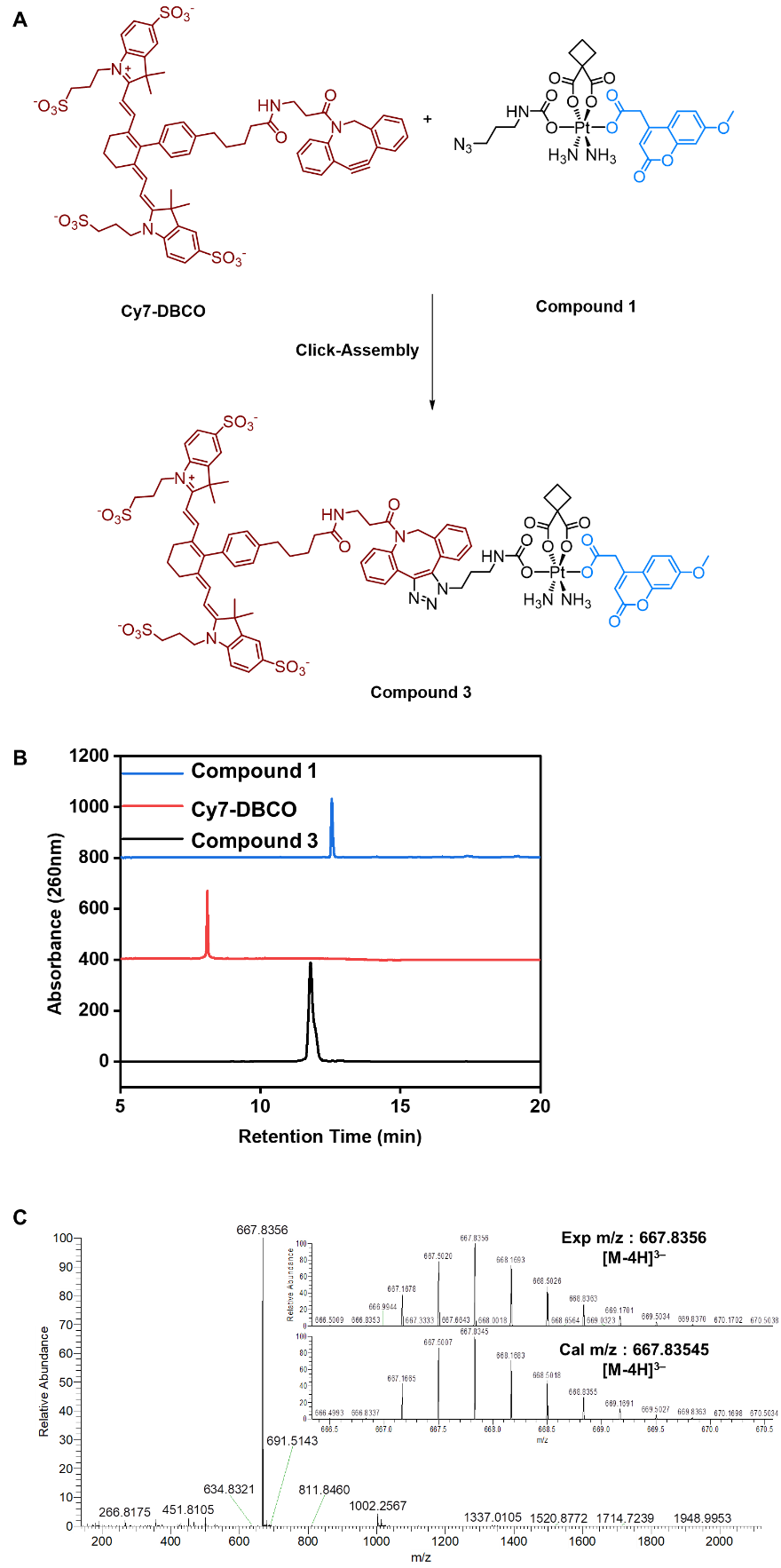


Fig S5. Characterization of Compound 3: **A**. Formation of Compound **3** via SPAAC click reaction between compounds **1** and **Cy7–DBCO**; **B**. HPLC Analysis; **C**. High resolution ESI–MS spectrum.


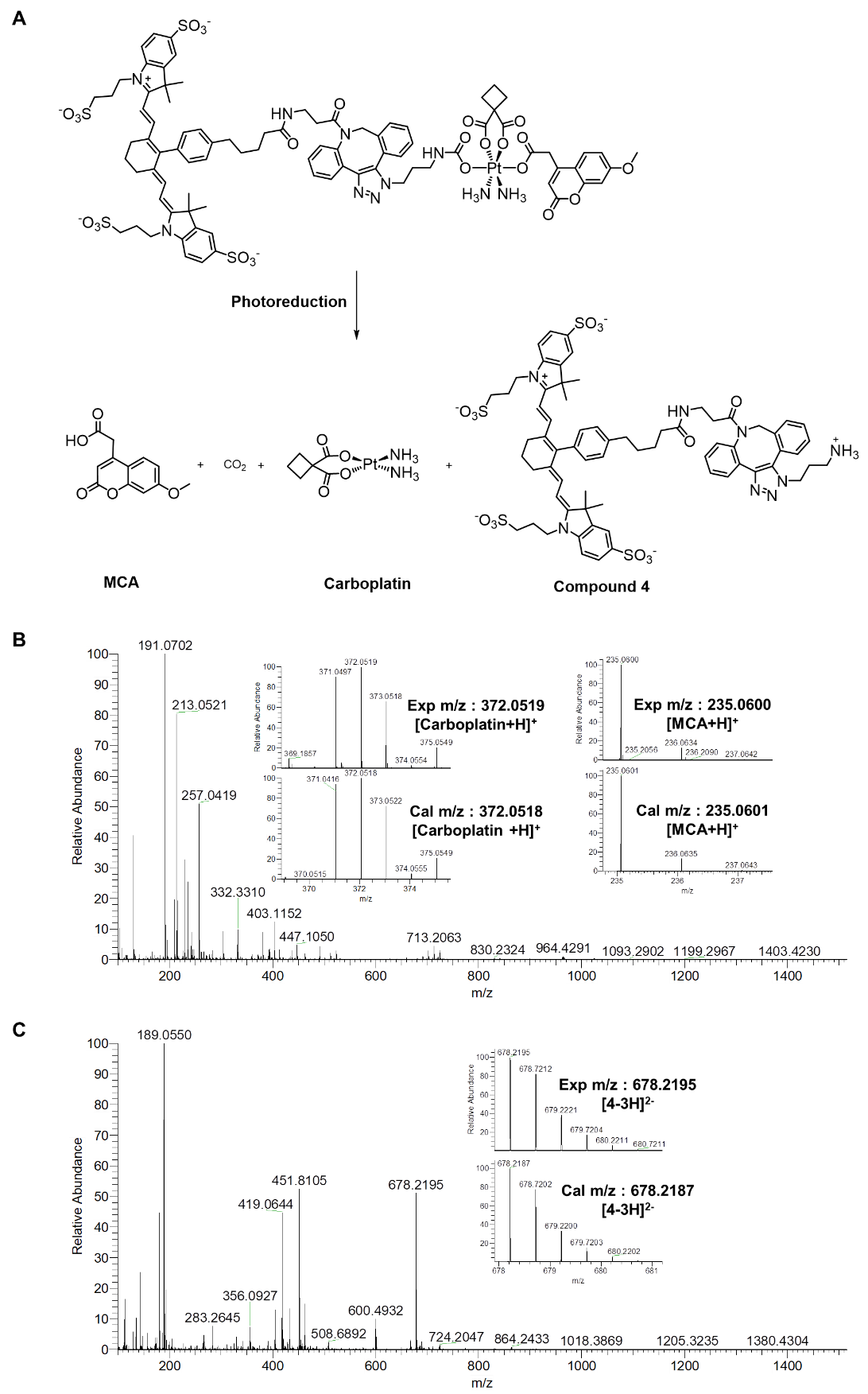


**Fig S6.** Photoreduction of Compound **3**: **A**. NIR irradiation induces the photoreduction of Compound **3**, yielding **MCA**, CO₂, carboplatin, and compound 4. **B**. High–resolution ESI–MS spectra in positive ion mode of the photoreduction products. **C**. High–resolution ESI–MS spectra in negative ion mode of the photoreduction products.


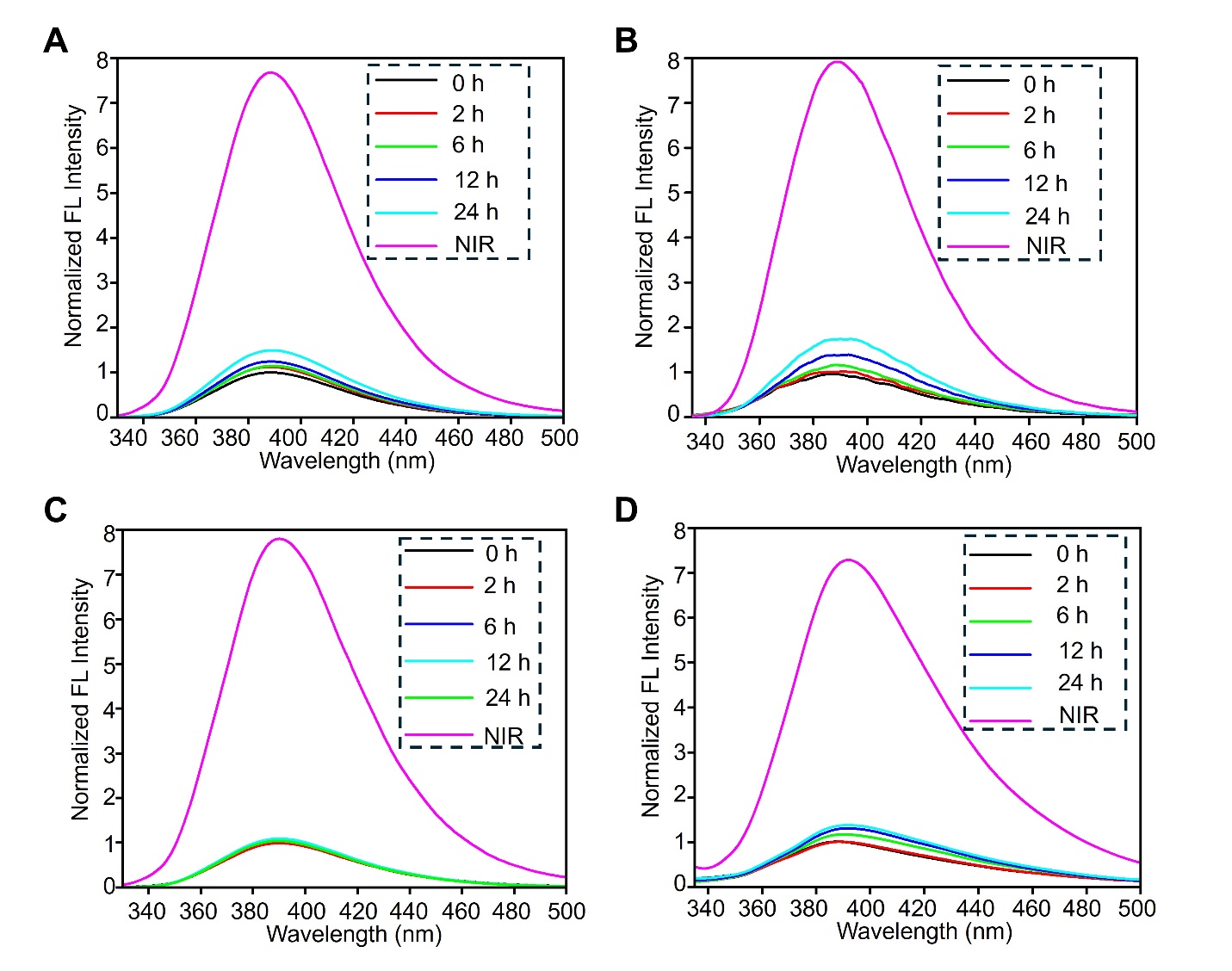


**Fig S7.** Fluorescence-based stability analysis of Compound **3** against biological reductants and in cell culture medium: **A**. Fluorescence spectra of **3** (5 µM) in PBS with ascorbic acid (100 µM) ± NIR irradiation (2 min, 830-nm LED). **B**. Fluorescence spectra of **3** (5 µM) in PBS with ascorbic acid (1 mM) ± NIR irradiation. **C**. Fluorescence spectra of **3** (5 µM) in PBS with glutathione (1 mM) ± NIR irradiation. **D**. Fluorescence spectra of **3** (5 µM) in cell culture medium ± NIR irradiation.


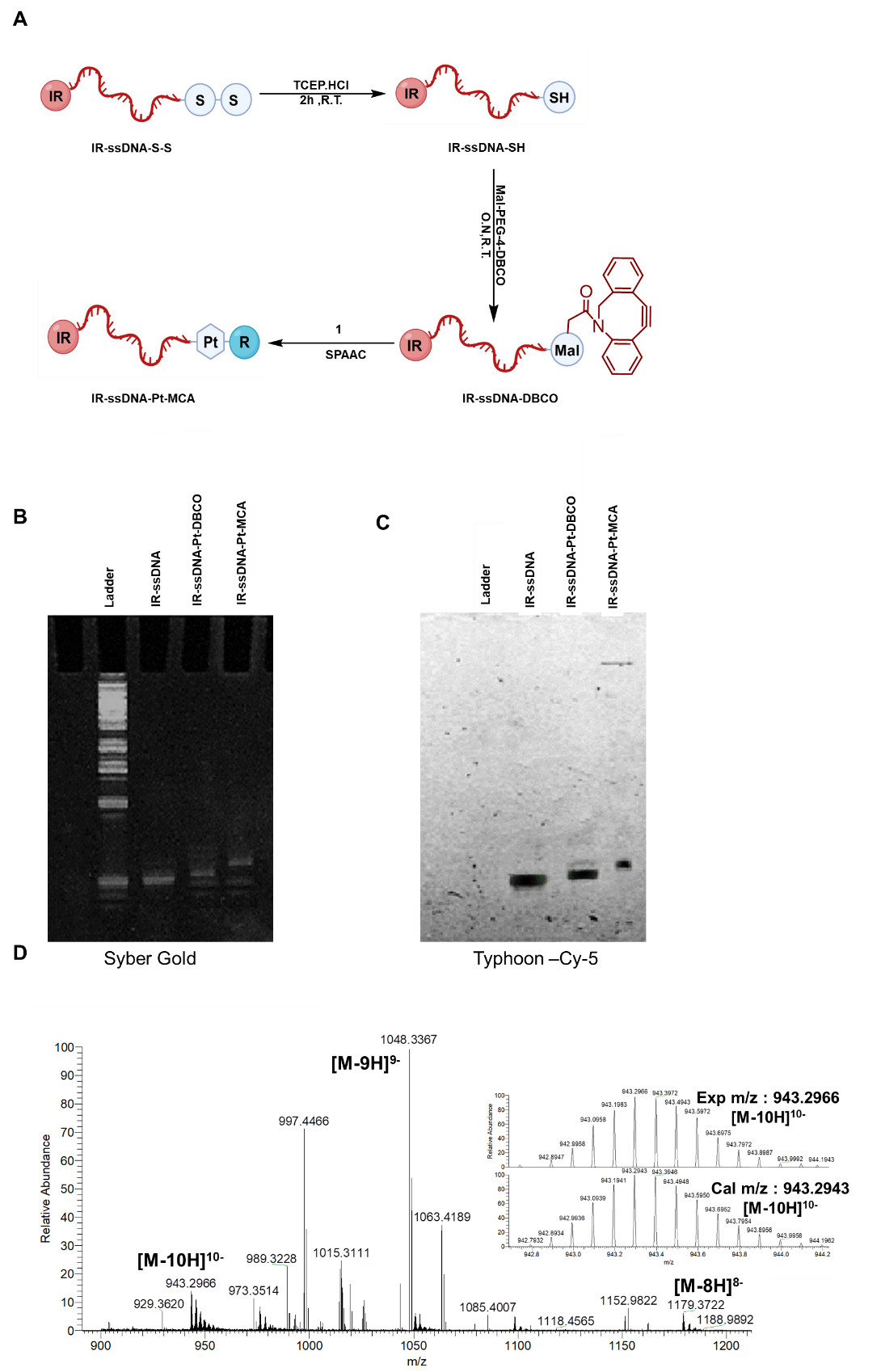


**Fig S8.** Synthesis and Characterization of **IR–ssDNA–Pt–MCA A.** Synthetic route for preparing **IR–ssDNA–Pt–MCA,** purified **IR–ssDNA–Pt–MCA** revealed by 18% denaturing PAGE gel. **B.** SYBR Gold: Lane 1: Low Molecular Weight DNA Ladder (NEB); Lane 2: IR–ssDNA; Lane 3: IR–ssDNA–DBCO; Lane 4: Purified click product (IR–ssDNA–Pt–MCA) **C.** Typhoon Imaging of the gel in Cy–5 (650 nm) filter Lane 2: IR–ssDNA; Lane 3: IR–ssDNA–DBCO; Lane 4: Purified click product (IR–ssDNA–Pt–MCA). **D**. High resolution ESI–MS spectrum.


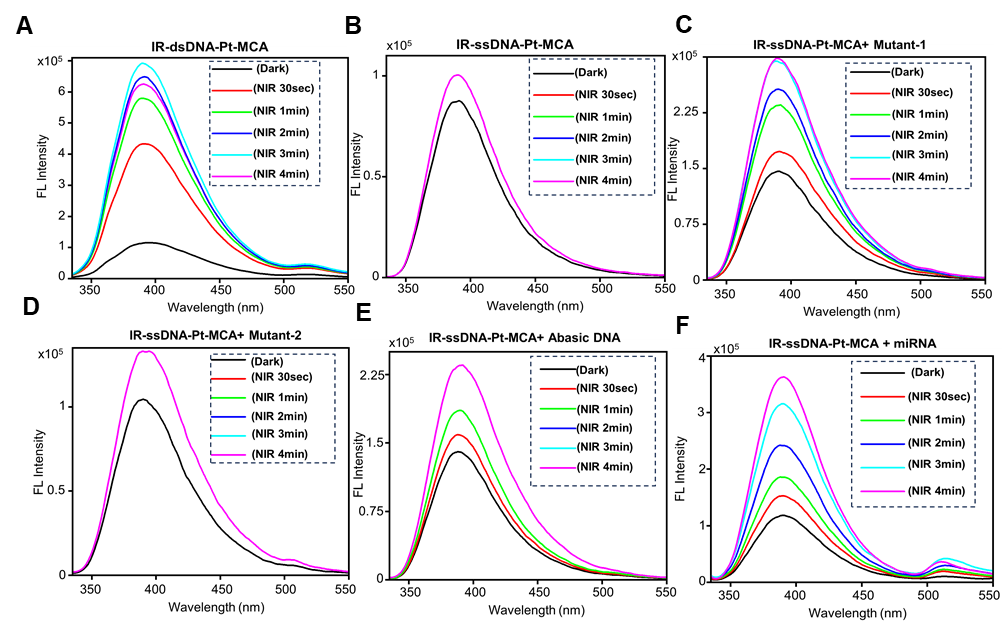


**Fig S9.** Fluorescence studies of **IR–ssDNA-Pt-MCA** with different **DNA variants**. **A.** Fluorescence studies of **IR–dsDNA-Pt-MCA. B.** Fluorescence studies of **IR–ssDNA-Pt-MCA. C.** Fluorescence studies of **IR–dsDNA-Pt-MCA + Mutant-1. D.** Fluorescence studies of **IR–dsDNA-Pt-MCA + Mutant-2. E.** Fluorescence studies of **IR–dsDNA-Pt-MCA + Abasic DNA. F.** Fluorescence studies of **IR–dsDNA-Pt-MCA + miRNA.**

**
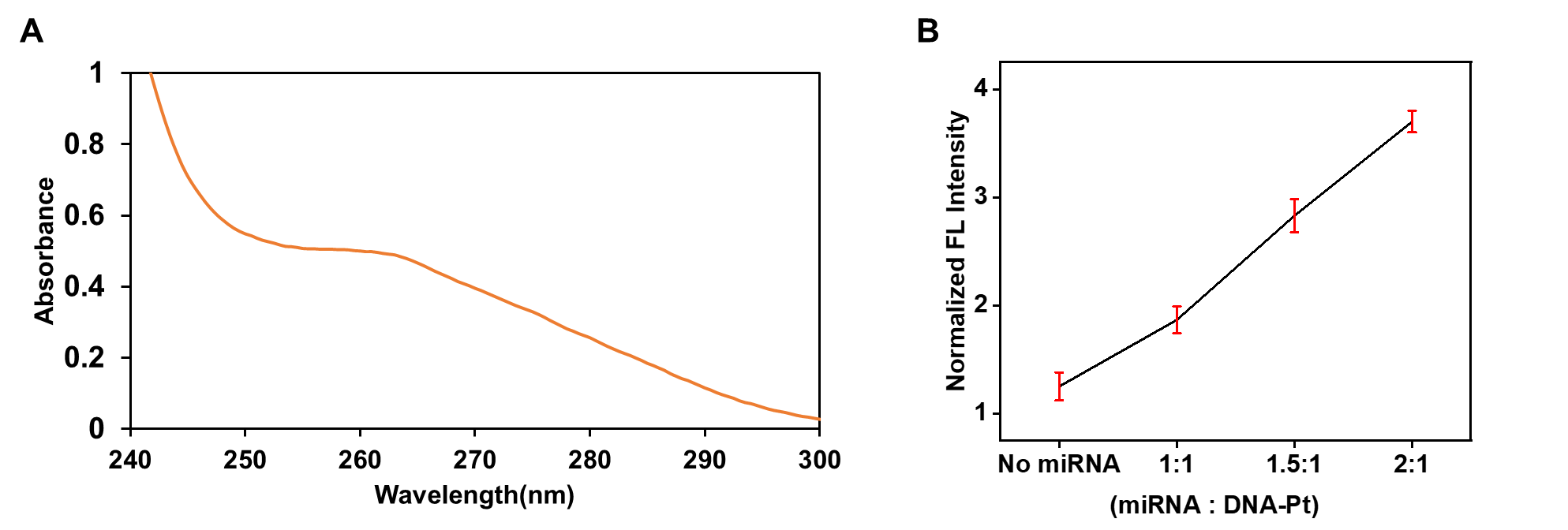
**

**Fig S10. miRNA extraction assay A.** RNA isolated using the PureLink™ miRNA Isolation Kit has an **OD_260/280_** of **1.955. B.** Fluorescence studies of IR–ssDNA–Pt–BDP + miRNA (from cells).


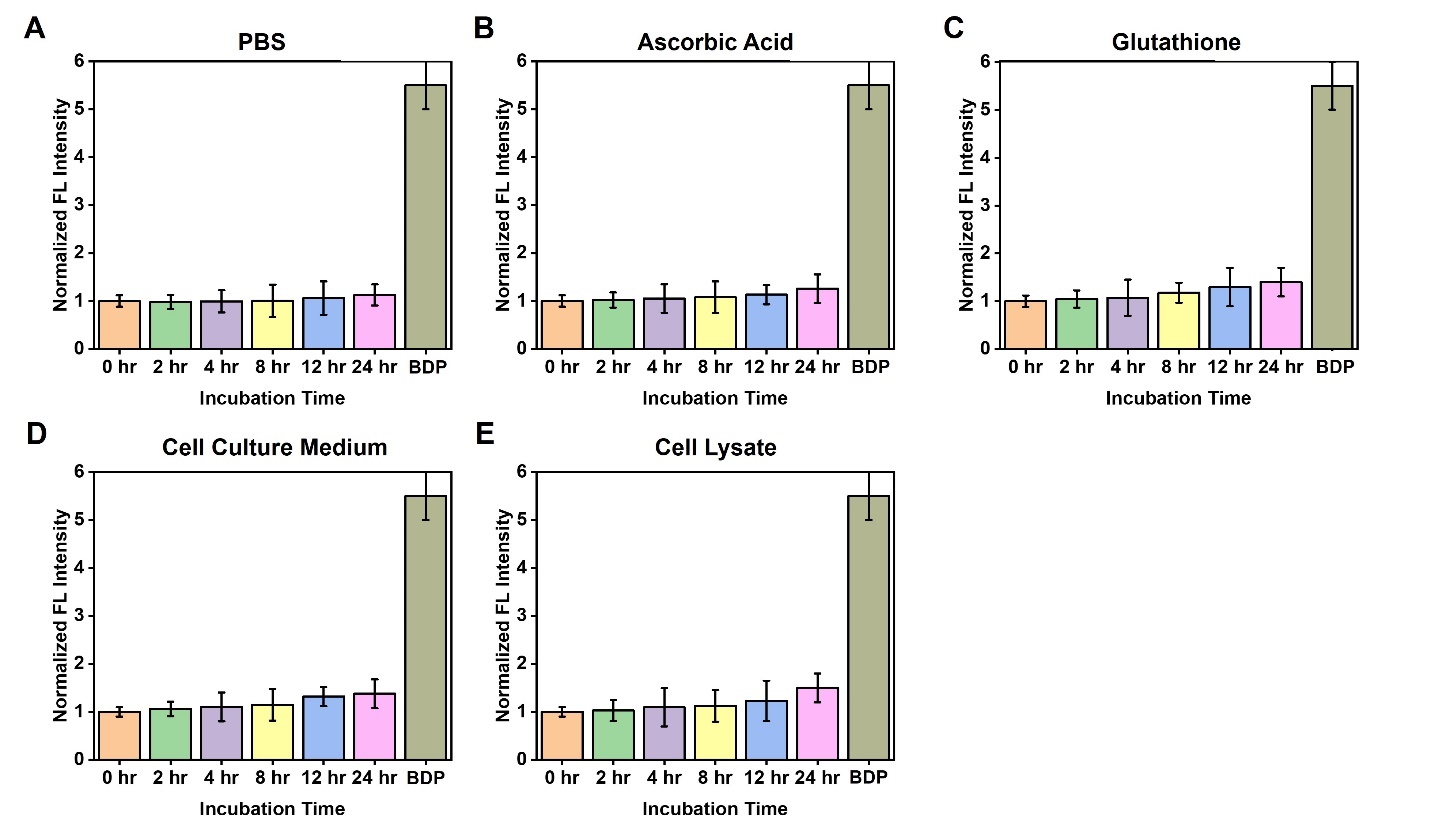


**Fig S11.** Fluorescence-based stability assessment of Compound **2** (5 µM) in the dark across various environments: **A.** PBS (pH = 7.4) over 24 h with free BDP reporter as control. **B**. PBS with ascorbic acid (500 µM). **C**. PBS with glutathione (6 mM). **D**. cell culture medium (DMEM (1 g/L glucose) supplemented with 10% FBS and 1% penicillin–streptomycin). **E**. Hela cell lysate. Data are shown as normalized fluorescence intensity.


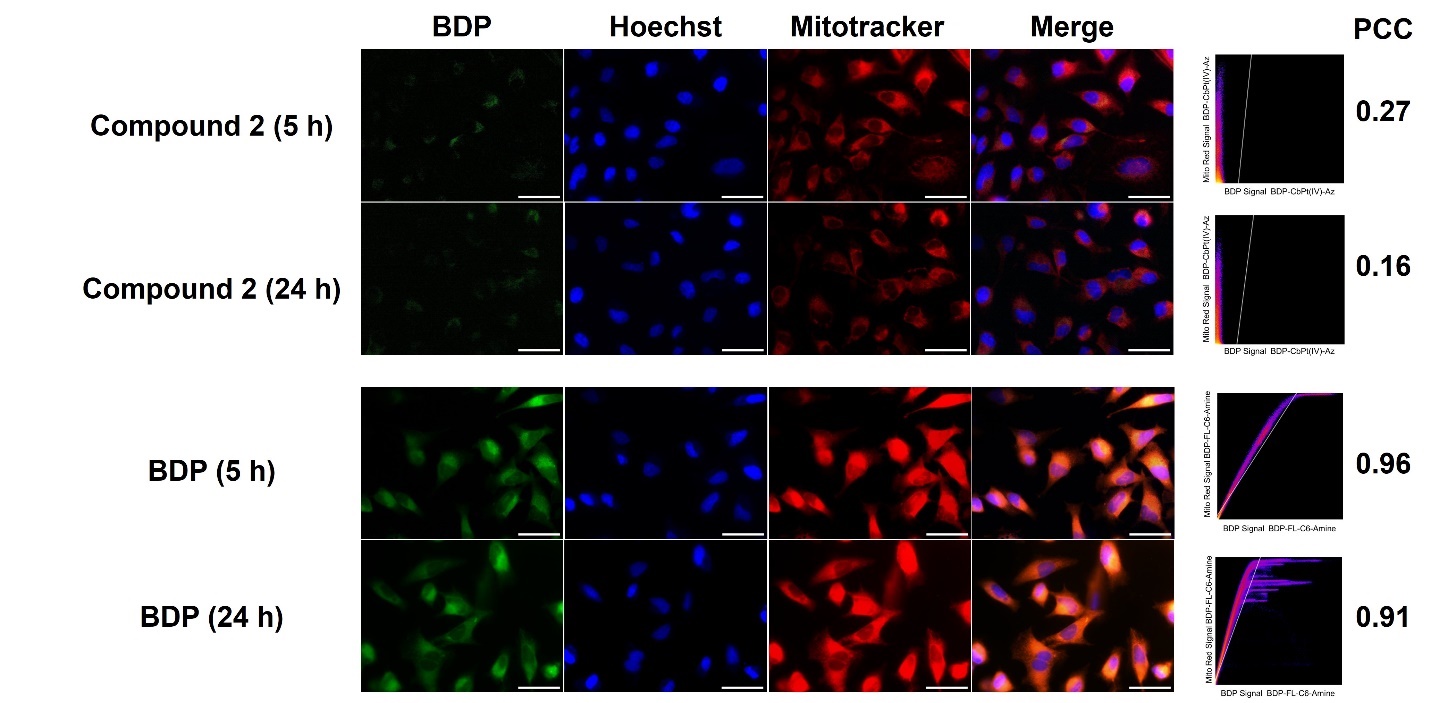


**Fig S12.** Imaging-based stability assessment of Compound **2** in the dark *in vitro* through time-dependent mitochondrial colocalization analysis. HeLa cells were incubated with Compound **2** or the free BDP reporter (5 µM) for 5 h or 24 h, followed by staining with MitoTracker Red (0.5 µM, for mitochondria staining) and Hoechst 33342 (2 µM, for nuclei staining. See “Cell imaging for assessing stability of Compound **2** in the dark *in vitro”* for detailed staining process). Live-cell fluorescence imaging was performed, and colocalization between BDP (green) and mitochondria (red) was quantified using ImageJ. Pearson’s correlation coefficients (PCC) and corresponding colocalization scatter plots are shown on the right. Scale bars: 50 µm. It is clear that compared with Compound **2**, BDP is more enriched in mitochondria, indicating negligible premature intracellular reduction of Compound **2** within the experimental timeframe.


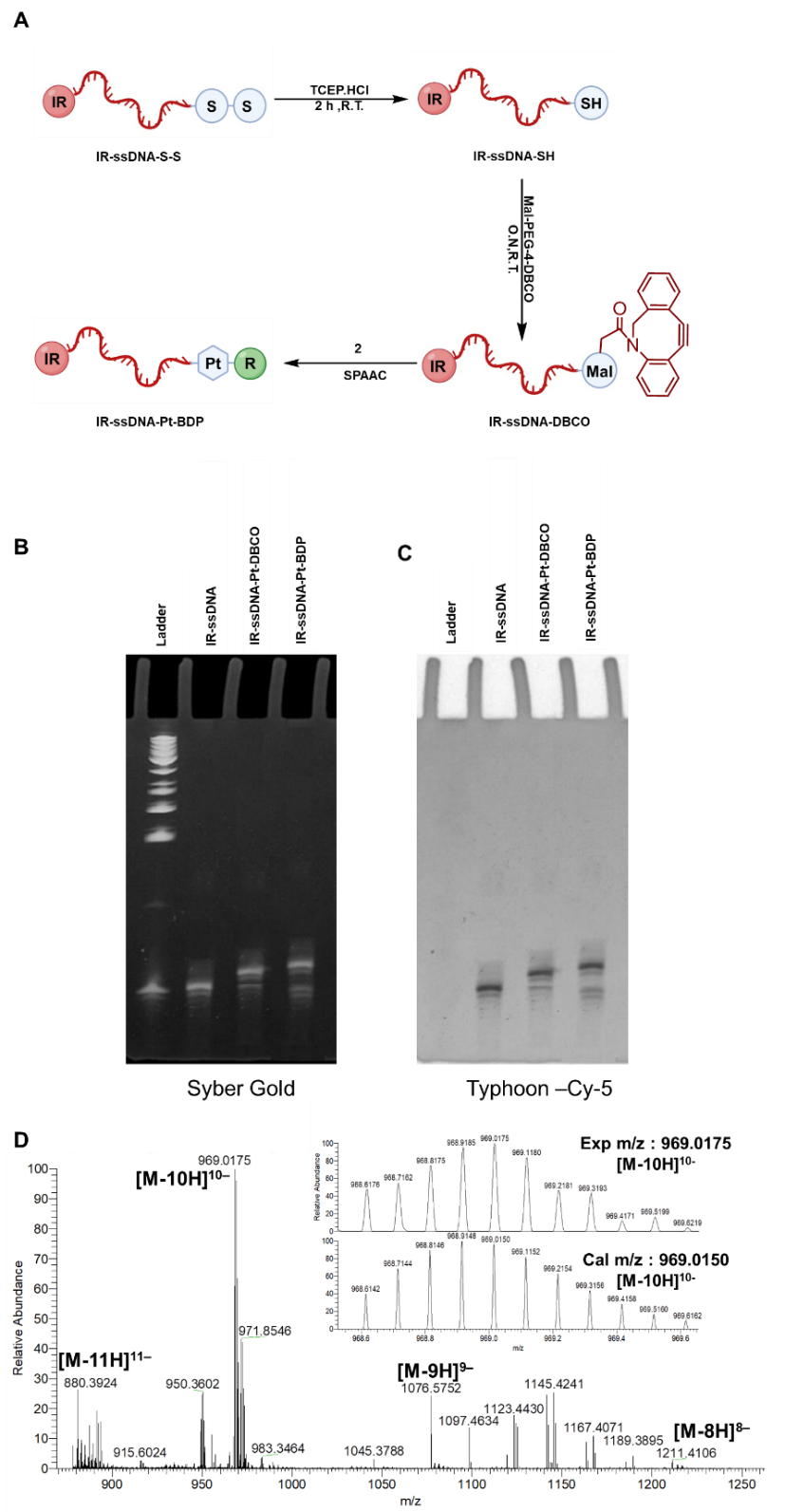


**Fig S13.** Synthesis and Characterization of **IR–ssDNA–Pt–BDP. A.** Synthetic route for preparing **IR–ssDNA–Pt–BDP**, purified IR–ssDNA–Pt–BDP revealed by 18% denaturing PAGE gel. **B.** SYBR Gold: Lane 1: Low Molecular Weight DNA Ladder (NEB); Lane 2: IR–ssDNA; Lane 3: IR–ssDNA–DBCO; Lane 4: Purified click product (IR–ssDNA–Pt–BDP) **C.** Typhoon Imaging of the gel in Cy–5 (650 nm) filter Lane 2: IR–ssDNA; Lane 3: IR–ssDNA–DBCO; Lane 4: Purified click product (IR–ssDNA–Pt–BDP). **D**. High resolution ESI–MS spectrum.


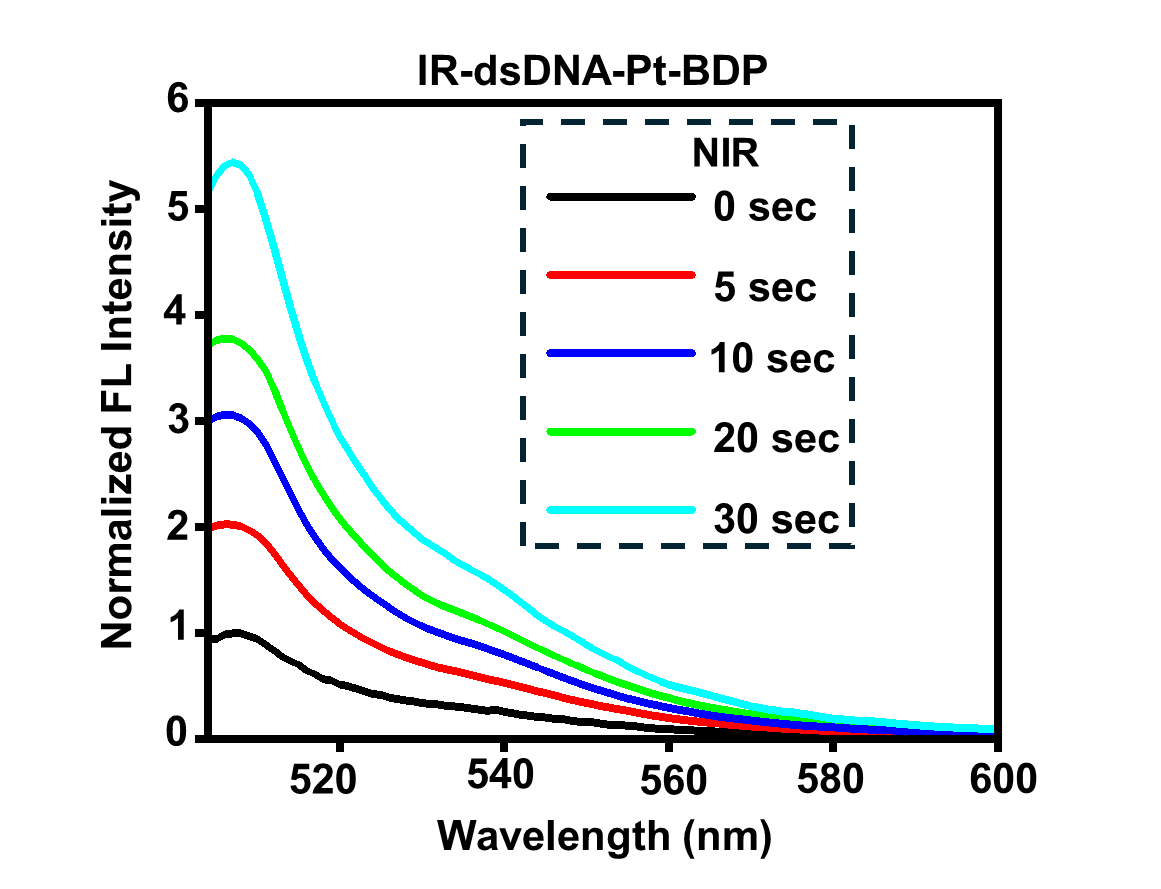


**Fig S14.** Fluorescence studies of **IR–dsDNA–Pt–BDP** before and after the irradiation.


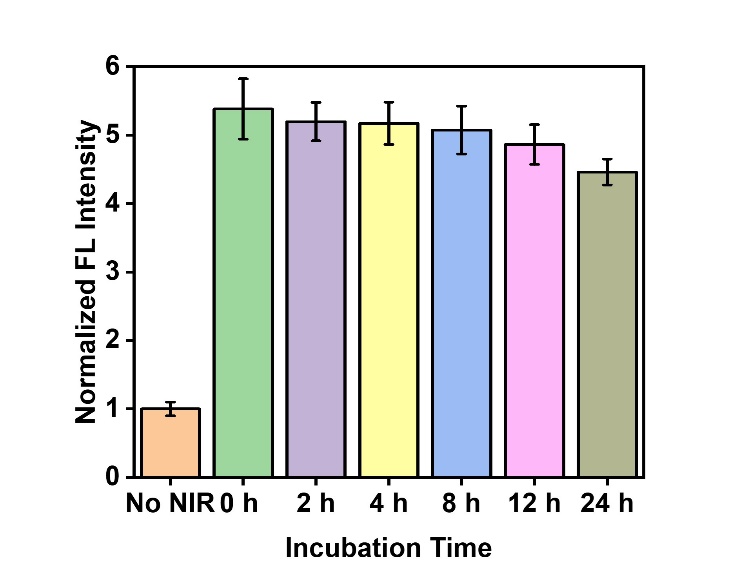


**Fig S15.** Fluorescence-based assessment of **IR–dsDNA–Pt–BDP** stability and NIR-triggered functionality in cell culture medium (DMEM (1 g/L glucose) supplemented with 10% FBS and 1% penicillin–streptomycin) over time. The construct was incubated in complete cell culture medium for varying durations (0–24 h), followed by NIR irradiation. Fluorescence turn-on from released BDP was measured to determine whether the construct retained its ability to undergo NIR-triggered activation and reporter release after incubation. A sample without NIR irradiation served as the control. It is clear that ~17% decrease in the **IR–dsDNA–Pt–BDP** sample was observed during 24-h incubation time.

**
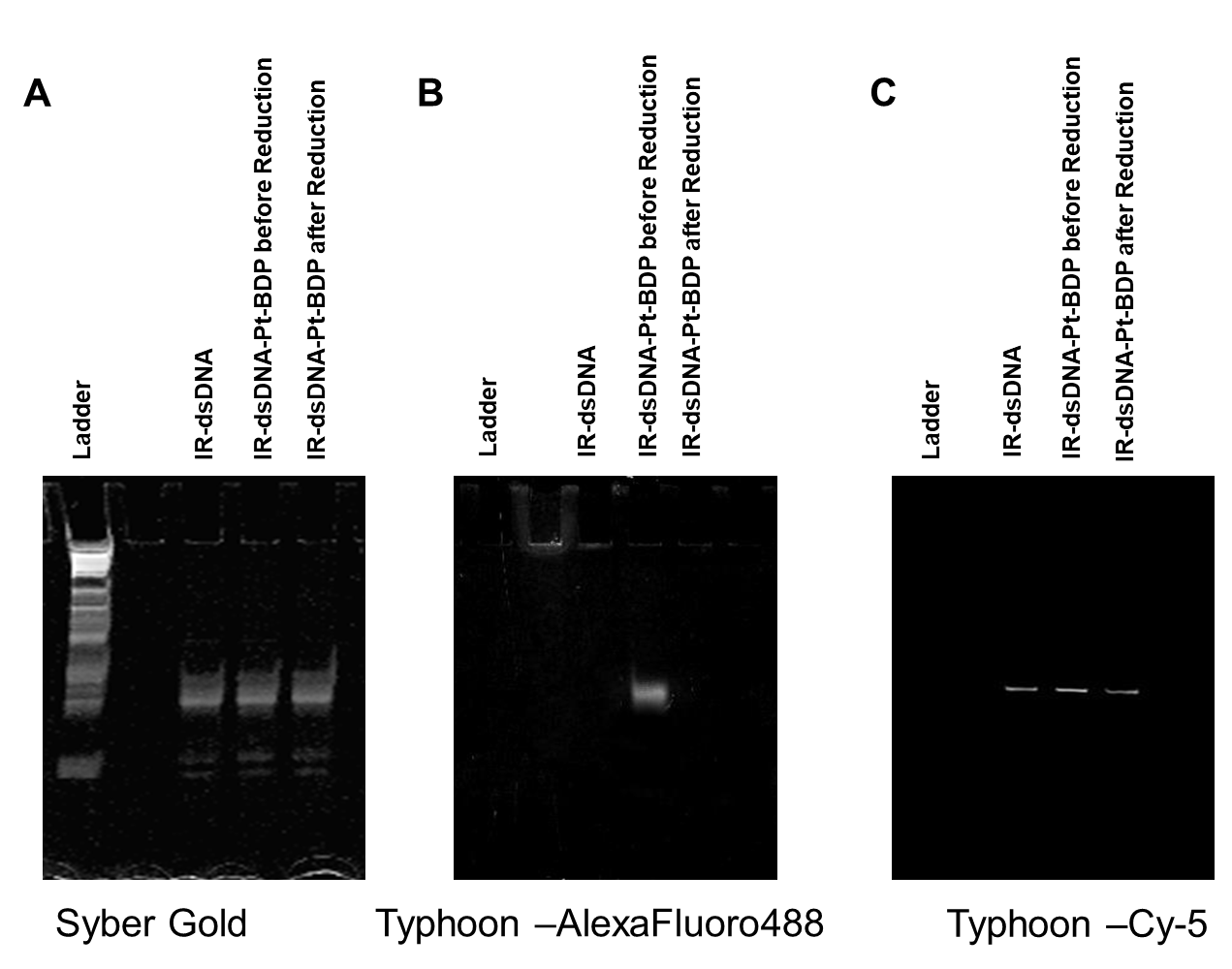
**

**Fig S16.** Photoreduction of **IR–dsDNA–Pt–BDP** using **PAGE gel–based assay**: **A**. SYBR Gold: Lane 1: Low Molecular Weight DNA Ladder (NEB); Lane 2: IR–dsDNA; Lane 3: IR–dsDNA–Pt–BDP before irradiation; Lane 4: IR–dsDNA–Pt–BDP after irradiation **B**. Typhoon Imaging of the gel in Typhoon–AlexaFluoro488 Lane 2: IR–dsDNA; Lane 3: IR–dsDNA–Pt–BDP before irradiation; Lane 4: IR–dsDNA–Pt–BDP after irradiation **C**. Typhoon Imaging of the gel in Cy–5 (650 nm) filter Lane 2: IR–dsDNA; Lane 3: IR–dsDNA–Pt–BDP before irradiation; Lane 4: IR–dsDNA–Pt–BDP after irradiation.

**Table S1. DNA Sequences:**

**IR–ssDNA:** 5’–/ 5IRD800/TTC AAC ATC AGT CTG ATA AGC TA/3ThioMC3–D/–3’

**Complementary DNA:** 5’–GTA GCT TAT CAG ACT GAT GTT GA –3’

**Mutant–1:** 5’–TA GCT TAT CAG ACT GAT ATT GA–3’

**Mutant–2:** 5’–TA GCT TAT CAG ACT GA**C AC**T GA–3’

**Abasic DNA:** 5’–TA GCT TAT CAG /idSp/CT GAT GTT GA–3’

**miRNA–21:** 5’–rUrArGrCrUrUrArUrCrArGrArCrUrGrArUrGrUrUrGrA–3’

**General information**. All reagents were purchased from Strem, Aldrich or Alfa and used without further purification. DBCO–containing dye was purchased from Lumiprobe Corporation and Vector Laboratories. DNA oligos were purchased from the Integrated DNA Technologies (IDT, IA). All reactions were carried out under normal atmospheric conditions. ¹H NMR spectra were acquired using a Bruker 400 MHz spectrometer. ¹³C NMR (125 MHz) and ¹⁹⁵Pt NMR (108 MHz) spectra were recorded using an Agilent 500 MHz spectrometer. Chemical shifts in ^1^H and ^13^C{^1^H} NMR spectra were internally referenced to solvent signals (^1^H NMR: DMSO–d_6_ at δ = 2.50 ppm; ^13^C NMR: DMSO–d_6_ at δ = 40.45 ppm). The ¹⁹⁵Pt NMR chemical shifts are referenced to an external standard of K₂PtCl₄ in D₂O at 23.7 °C. The high–resolution mass spectra of created ions were recorded on an Exactive Plus mass spectrometer (Thermo Scientific, Bremen, Germany). The absorption spectra of compound 3 and miRNA were recorded using a Thermo Fischer Scientific Nanodrop one. Fluorescence spectra were recorded using a FluoroMax–3 fluorescence spectrophotometer using the software called FluorEssence. GFAAS measurements were taken on a PerkinElmer PinAAcle 900Z spectrometer. Fluorescence images were acquired using an Olympus IX70 inverted epifluorescence microscope equipped with a digital CCD camera (QImaging). Images were processed and intensities were quantified with ImageJ software (Fuji). Analytical HPLC was conducted on an Agilent 1100 system (Agilent ZORBAX Eclipse XDB–C18 reverse-phase column (5 µm, 4.6 x 150 mm), mobile phase: solvent A is 0.1% TFA aqueous solution and B is acetonitrile, Flow Rate; 1 mL/min).

Synthesis of CarboPt(IV)–(OH)_2_: **Carboplatin** (100 mg, 0.27 mmol) was suspended in 2.5 mL of double–deionized water in a round–bottom flask at room temperature, followed by the addition of 0.5 mL of 35% H₂O₂. The reaction mixture was stirred at room temperature for 24 h. After completion, the water was removed by evaporation under reduced pressure. The crude product was resuspended in methanol, followed by the addition of diethyl ether, resulting in the formation of a white precipitate. The supernatant was removed by centrifugation, and the product was washed twice with diethyl ether. The final product was dried overnight in a desiccator. Yield: 85.5 mg, 78.1%.

Synthesis of CarboPt(IV)–MSC: In a 6–dram glass vial, **CarboPt(IV)–(OH)₂** (50 mg, 0.123 mmol, 1 equiv.) and DSC (22.12 mg, 0.0864 mmol, 0.7 equiv.) were added. Under a nitrogen atmosphere, anhydrous DMF (2 mL) was added, and the mixture was stirred for 1 h at room temperature. The reaction mixture was evaporated to dryness under reduced pressure, and the resulting crude product was redissolved in 0.7 mL acetonitrile and precipitated with diethyl ether. The precipitate was collected by centrifugation, and the supernatant was discarded. The solid product was washed twice with 2 mL diethyl ether and dried overnight in a desiccator. Yield: 43.7 mg, 95%. ^1^H NMR (500 MHz, DMSO–d_6_) δ 6.14 – 5.54 (m, 6H), 2.69 (s, 4H), 2.52 (t, 4H), 1.78 (p, *J* = 8.1 Hz, 2H). ^13^C NMR (126 MHz, DMSO–d_6_) δ 176.79, 173.26, 171.24, 162.78, 154.32, 56.09, 40.47, 40.30, 40.13, 39.96, 39.80, 39.63, 39.46, 25.69, 16.20. ^195^Pt NMR (108 MHz, DMSO–d_6_) δ 1808.88. HR-ESI-MS: [M+Na]^+^: calcd for 569.0457, found: 569.0455

Synthesis of CarboPt(IV)–Az–O: In a 6–dram glass vial 3–Azidopropyl–1–amine (29.32 mg, 28.75 μL, 0.293 mmol, 4equiv) was dissolves in DMF(1 mL) under a nitrogen atmosphere followed by addition of DIPEA(189.35mg, 255.19 μL, 1.465mmol, 20equiv), and the reaction mixture was stirred for 20 min at room temperature. In a separate vial **CarboPt(IV)–MSC** (40.00 mg, 0.0732 mmol, 1equiv) was dissolve in anhydrous DMF (0.50 mL) and the solution was transferred to the reaction mixture under nitrogen atmosphere and was stirred overnight in the dark. The solvent was removed under reduced pressure using a rotary evaporator. To the resulting residue, acetone (2.5 mL) was added, and the mixture was sonicated until a solid precipitate formed. The precipitate was collected by centrifugation, and the brown supernatant was discarded. This washing process with acetone was repeated twice. The precipitate was then washed twice with diethyl ether (1.5 mL each) and dried overnight in a desiccator. Yield: 31 mg, 83% ^1^H NMR (500 MHz, DMSO–d_6_) δ 6.28 (t, *J* = 6.4 Hz, 1H), 6.19 – 5.77 (m, 6H), 3.31 (t, *J* = 6.8 Hz, 4H), 2.95 (q, *J* = 6.4 Hz, 2H), 2.66 (t, *J* = 7.9 Hz, 2H), 1.77 (p, *J* = 8.0 Hz, 2H), 1.59 (p, 2H). ^13^C NMR (126 MHz, DMSO–d_6_) δ 177.98, 177.02, 171.58, 164.58, 162.78, 56.12, 56.00, 48.98, 48.83, 40.46, 40.30, 40.13, 39.96, 39.79, 39.63, 39.46, 36.25, 30.79, 29.73, 28.88, 16.33, 15.45. ^195^Pt NMR (108 MHz, DMSO–d_6_) δ 1782.77. HR-ESI-MS: : [M+Na]^+^: calcd for 532.1118, found: 532.1114.

Synthesis of Compound 1: In a 6–dram glass vial, **CarboPt(IV)–Az–O** (30.0 mg, 0.0565 mmol, 1 equiv.) and 7–Methoxycoumarin–4–acetic acid (14.54 mg, 0.0621 mmol, 1.1 equiv.) were combined in anhydrous DMSO (2.5 mL) under a nitrogen atmosphere. TBTU (27.21 mg, 0.0848 mmol, 1.5equiv.) and DIPEA (10.95 mg, 14.76 μL, 0.0848 mmol, 1.5equiv.) were added and the reaction mixture was stirred at 50 °C for 12h. The reaction mixture was lyophilized, and the resulting crude product was redissolved in 1.5 mL MeOH:DCM (50:50 by volume) and was sonicated. The precipitate was removed by centrifugation and the HPLC purification was performed on supernatant to obtain the product. Yield: 21.1 mg, 50%  ^1^H NMR (400 MHz, DMSO–d_6_) δ 7.71 (d, *J* = 8.8 Hz, 1H), 6.98 (d, *J* = 2.6 Hz, 1H), 6.89 (dd, *J* = 2.6, 9.0 Hz, 1H), 6.63 (t, *J* = 5.3 Hz, 1H), 6.25-6.61 (m, 6H), 6.23 (s, 1H), 3.86 (s, 3H), 3.83 (s, 2H), 3.32 (t, *J* = 7.0 Hz, 2H), 2.97 (q, *J* = 6.3 Hz, 2H), 2.47 (q, *J* = 8.4 Hz, 4H), 1.70-1.95 (m, *J* = 8.0 Hz, 2H), 1.45-1.70 (m, *J* = 7.2 Hz, 2H). ^13^C NMR (126 MHz, DMSO–d_6_) δ 176.59, 163.60, 162.67, 160.70, 158.19, 155.36, 151.34, 127.92, 113.21, 113.03, 112.50, 101.03, 56.37, 55.86, 48.86, 40.85, 38.59, 31.95, 31.41, 29.54, 16.17. ^195^Pt NMR (108 MHz, DMSO–d_6_) δ 1978.85. HR-ESI-MS: [M+Na]^+^: calcd for [C_22_H_28_N_6_O_11_Pt+Na]^+^: 770.1357, found: 770.1358. Purity: 98% (determined by HPLC).

Synthesis of CarboPt(IV)–COOH**:** In a 6-dram glass vial, **CarboPt(IV)-(OH)₂** (171 mg, 0.42 mmol, 1 equiv.) and succinic anhydride (42.2 mg, 0.42 mmol, 1 equiv.) were added. Under a nitrogen atmosphere, anhydrous DMSO (2 mL) was added, and the mixture was stirred overnight at room temperature. The reaction mixture was lyophilized, and the resulting crude product was redissolved in 3 mL methanol and precipitated with diethyl ether. The precipitate was collected by centrifugation, and the supernatant was discarded. The solid product was washed twice with 5 mL diethyl ether and dried overnight in a desiccator. Yield: 170 mg, 80%.

Synthesis of CarboPt(IV)–Azide**:** In a 6–dram glass vial, **CarboPt(IV)–COOH** (147.2 mg, 0.291 mmol, 1 equiv.) and HATU (121 mg, 0.32 mmol, 1.1 equiv.) were combined. Under a nitrogen atmosphere, anhydrous DMF (2 mL) was added, and the mixture was stirred for 20 min at room temperature. 3–Azido–1–propanamine (73.6 µL, 0.727 mmol, 2.5 equiv.) was added, and stirring was continued for another 20 min in the dark. DIPEA (256 µL, 1.45 mmol, 4 equiv.) was then added dropwise, and the reaction mixture was stirred overnight in the dark. The solvent was removed under reduced pressure using a rotary evaporator. To the resulting residue, acetone (2.5 mL) was added, and the mixture was sonicated until a solid precipitate formed. The precipitate was collected by centrifugation, and the brown supernatant was discarded. This washing process with acetone was repeated twice. The precipitate was then washed twice with diethyl ether (3 mL each) and dried overnight in a desiccator. Yield: 110 mg, 64%. ^1^H NMR (400 MHz, DMSO-d_6_) δ 7.88 (t, 1H, J = 5.6 Hz), 5.68-6.4 (m, 6H), 3.34 (t, 2H, J = 6.7 Hz), 3.08 (q, 2H, J = 6.4 Hz), 2.5-2.6 (m, 4Hz), 2.2-2.42 (m, 4H,), 1.74-1.86 (m, 2H, J = 8.2 Hz), 1.55-1.7 (m, 2H, J = 6.8 Hz). ^13^C NMR (125 MHz, DMSO-d_6_): 180.0, 177.0, 172.1, 56.1, 48.9, 36.3, 32.7, 32.0, 31.3, 28.9, 16.3. ^195^Pt NMR (108 MHz, DMSO-d_6_) δ 1760.22. HR-ESI-MS: [M+Na]^+^: calcd for [C_13_H_24_N_6_NaO_8_Pt]^+^: 610.1192, found: 610.1196.

Synthesis of MSC–CarboPt(IV)–Azide**:** Conversion of the axial hydroxide (OH) ligand to a carbonate (MSC) linkage is modified from the procedure previously reported by Gibson.^1^ In a 6–dram glass vial, **CarboPt(IV)–Azide** (45 mg, 0.076 mmol, 1 equiv.) and N,N'–disuccinimidyl carbonate (DSC) (30 mg, 0.11 mmol, 1.5 equiv.) were mixed, and anhydrous DMF was added under a nitrogen atmosphere. The reaction mixture was stirred overnight at room temperature. The solvent was removed under reduced pressure using a rotary evaporator. To the residue, acetonitrile (1 mL) followed by diethyl ether (10 mL) was added, resulting in the formation of a white precipitate. The precipitate was collected by centrifugation, and the supernatant was discarded. The solid was washed twice with diethyl ether (3 mL each) and dried overnight in a desiccator. Yield: 98% (54 mg). ^1^H NMR (400 MHz, DMSO-d_6_): 7.93 (t, 1H, J = 5.6 Hz), 6-6.65 (m, 6H), 3.34 (t, 2H, J = 6.9 Hz), 3.08 (q, 2H, J = 6.3 Hz), 2.71 (s, 4H), 2.48-2.58 (m, 4H, J =, 7.7 Hz), 2.21-2.4 (m, 4H, J = 7.7 Hz), 1.75-1.88 (m, 2H, J = 8.1 Hz), 1.55-1.75 (m, 2H, J = 6.8 Hz). ^13^C NMR (125 MHz, DMSO-d_6_): 179.4, 176.5, 171.6, 171.0, 153.7, 55.9, 48.9, 36.3, 33.0, 31.5, 30.6, 30.1, 25.7, 16.2. ^195^Pt NMR (108 MHz, DMSO-d_6_) δ 2004.1 HR-ESI-MS: [M+H]^+^: calcd for [C_18_H_27_N_7_O_12_Pt]^+^: 729.1442, found: 729.1440.

Synthesis of Compound 2**:** **MSC–CarboPt(IV)–Azide** (30 mg, 0.041 mmoles, 3equiv.) and Bodipy Amine ( 5.75 mg, 0.013 mmoles, 1equiv.) were combined to a 20 mL glass vial. Anhydrous DMF (1.5 mL) was added under a nitrogen atmosphere and the mixture was stirred for 20 min at room temperature. DIPEA (9 µL, 0.052 mmoles, 4 equiv.) was then added, and the reaction mixture was stirred overnight at room temperature. The solvent was concentrated under reduced pressure using a rotary evaporator. The crude product was purified by flash chromatography on silica gel (elution gradient: DCM:MeOH 0% to 20%). Yield: 10.5 mg, 52%. ^1^H NMR (400 MHz, DMSO–d_6_) δ 7.91 (t, 3H), 7.70 (s, 1H), , 6.45-6.62 (m, 6H), 6.51 (t, 1H), 6.25-6.40 (m, 2H), 3.41 (t, J = 6.9 Hz, 1H), 3.08 (q, J = 6.3 Hz, 4H)3.07 (d, *J* = 6.8 Hz, 1H), 2.42-2.54 (m, 4H, , 7.7 Hz), 2.47 (s, 6H), 2.2-2.3 (m, , 4H), 2.2-2.3 (m, 2H), 1.75-1.84 (m, , 2H), 1.63 (p, *J* = 6.7 Hz, 2H), 1.36 – 1.19 (m, 8H). ^13^C NMR (125 MHz, DMSO-d_6_) δ 194.38, 176.16, 171.53, 170.78, 163.39, 157.96, 144.26, 129.06, 125.45, 120.41, 116.70, 72.41, 69.87, 60.30, 55.52, 53.66, 48.52, 41.92, 38.61, 35.98, 33.84, 31.24, 29.88, 28.44, 26.34, 26.23, 25.32, 24.13, 18.15, 16.80, 15.80, 14.61, 12.56, 11.10. ^195^Pt NMR (108 MHz, DMSO-d_6_) δ 1973.59. Purity: 98% (determined by HPLC).

**Synthesis of IR–ssDNA–Pt–MCA:** In a PCR tube, **10 µL of DNA (100 µM)** was mixed with **2 µL of TCEP–HCl (1 mM)** and incubated for **2 h** to reduce disulfide (S–S) bonds to free thiol (S–H) groups. Following this, **Maleimide–PEG–4–DBCO** was added in a **1:20 ratio** (where DNA was considered as 1 equivalent), and the reaction was allowed to proceed **overnight** to ensure efficient conjugation. The reaction mixture was then purified using **3K Amicon filters**, effectively removing excess Maleimide–DBCO. The purified DNA–DBCO conjugate was subsequently subjected to **strain–promoted azide–alkyne cycloaddition (SPAAC)** by reacting with Cou–O–CarboPt(IV)–Az–O in a **1:50 ratio,** followed by **overnight incubation** at room temperature. Finally, the DNA construct was purified using a **NAP–5 column**, ensuring the removal of any unreacted components and obtaining the desired modified DNA. The click product was analyzed with the 18% denature PAGE gel and the structure was confirmed by HR-ESI-MS: [M–10H]^–10^: calcd for [C_333_H_408_N_96_O_165_P_24_PtS_2_]^10–^: 943.2943 found: 943.2966

**Synthesis of IR–ssDNA–Pt–BDP:** In a PCR tube, **10 µL of DNA (100 µM)** was mixed with **2 µL of TCEP–HCl (1 mM)** and incubated for **2 h** to reduce disulfide (S–S) bonds to free thiol (S–H) groups. Following this, **Maleimide–PEG4–DBCO** was added in a **1:20 ratio** (where DNA was considered as 1 equivalent), and the reaction was allowed to proceed **overnight** to ensure efficient conjugation. The reaction mixture was then purified using **3K Amicon filters**, effectively removing excess Maleimide–DBCO. The purified DNA–DBCO conjugate was subsequently subjected to **strain–promoted azide–alkyne cycloaddition (SPAAC)** by reacting with BDP–CarboPt(IV)–Az in a **1:50 ratio,** followed by **overnight incubation** at room temperature. Finally, the DNA construct was purified using a **NAP–5 column**, ensuring the removal of any unreacted components and obtaining the desired modified DNA. The click product was analyzed with the 18% denature PAGE gel and the structure was confirmed by HR-ESI-MS: [M–10H]^–10^: calcd for [C_348_H_431_BF_2_N_100_O_164_P_24_PtS_2_]^10–^: 969.0175 found: 969.0150.

Click-Assembly of Photoactivatable Pt(IV) Compound (3)**:** **Compound 1** and Cy7-DBCOwere mixed in a 1:1 ratio in DMSO and were allowed to react via strain promoted azide-alkyne cycloaddition (SPAAC) at r.t.. After 2h the reaction mixture was directly purified by semi-preparative HPLC and fractions containing the desired product (Purity ≥95%) were collected which was lyophilized to get the final products. These Compounds were confirmed by HR-ESI-MS. HR-ESI-MS: [M-4H]^3-^: calcd for [C_87_H_95_N_10_O_25_PtS_4_]^3–^: 667.8356, found: 667.8345.

**Fluorescence studies of Compound 3 before and after NIR irradiation:** Fluorescence emission studies were conducted on Compound **3** before and after irradiation with NIR light. A FluoroMax–3 fluorescence spectrophotometer equipped with FluorEssence software was used to record the fluorescence spectra. Ascorbic acid (100 µM) and Compound **3** (9 µM) solutions were made in PBS (pH 7) at room temperature in cuvettes in a dark environment. Emission spectra were recorded at 0 min (in the dark) as the initial fluorescence measurements followed by after irradiation at different intervals. This detection step involved brief excitation at the coumarin absorption wavelength (in the UV–blue range), typically for only a few seconds during the measurement. The UV excitation was kept at low intensity and short duration to avoid inducing significant photoreduction by itself.

**Fluorescence Studies of Compound 3 in Response to LED Irradiation at Variable Wavelengths:** Photo–uncaging experiments were conducted to investigate the fluorescence changes of 3 using LED light sources at varying wavelengths (490, 590, 680, and 810 nm). Solutions of **3** (9 µM) and ascorbic acid (100 µM) were prepared in PBS (pH 7) at room temperature in cuvettes under dark conditions. Initial fluorescence measurements were recorded at 0 min (in the dark), followed by emission spectra collected at specified time intervals after irradiation (2 min).

**HPLC and ESI–MS analysis of Photo–Uncaging of Compound 3:** The photo–uncaging experiments were performed using an 830-nm LED light source (42.6 mW/cm²). A sample solution was prepared by mixing Pt Compounds (100 µM), ascorbic acid (200 µM), and water to a final volume of 350 µL in an HPLC vial. The sample was irradiated for a specified duration and analyzed using an HPLC system to obtain chromatograms. Upon completion of photo–uncaging, no peaks corresponding to the starting material were detected. The photo–released products were confirmed using high–resolution mass spectrometry (HR-ESI-MS).

**Fluorescence-based stability assessment of Compound 3 in the dark:** The stability of Compound **3** was evaluated against biological reductants and in cell culture medium using fluorescence spectroscopy. Each sample was prepared at a final concentration of 5 μM in PBS buffer (pH 7.4). For stability testing, ascorbic acid (100 μM and 1 mM), glutathione (1 mM), and a solution of complete DMEM medium (1 g/L glucose), were freshly prepared in PBS and mixed with the Compound **3**. The mixtures were incubated at 37 °C in the dark, and aliquots were withdrawn at predetermined time points (0 h, 2 h, 6 h, 12 h, and 24 h). Fluorescence intensity changes were recorded to monitor stability, with and without the NIR irradiation.

**Fluorescence studies of DNA–Pt–Scaffolds before and after NIR irradiation:** Fluorescence emission studies were conducted on IR–ssDNA–Pt–MCA, IR–dsDNA–Pt–MCA, IR–ssDNA–Pt–MCA annealed to Mutant –1 DNA, Mutant –2 DNA, Abasic DNA, miRNA before and after irradiation with NIR light. A FluoroMax–3 fluorescence spectrophotometer equipped with FluorEssence software was used to record the fluorescence spectra. Ascorbic acid (100 µM) and above–mentioned compounds (5 µM) solutions were made in PBS (pH 7) at room temperature in cuvettes in a dark environment. Emission spectra were recorded at 0 min (in the dark) as the initial fluorescence measurements followed by after irradiation at different intervals.

**Cell Culture:** Hela were obtained via American Type Culture Collection and was cultured in DMEM 1 g/L glucose, with L–glutamine with L–glutamine & sodium pyruvate (Corning) supplemented with 10% FBS and 1% Penicillin–Streptomycin (Corning). All cell lines were maintained at 37°C in a 5% CO_2_.Cells were passaged at 70–80% confluence by trypsinization and split in a 1:5 ratio.

**miRNA extraction assay:** miRNA, was isolated from the Hela cells by using PureLink™ miRNA Isolation Kit (Thermo Fischer, Waltham, MA, USA) and following a standardized protocol to ensure high–quality purification. Firstly, Hela cells (up to 1 × 10⁶) were harvested by removing the DMEM 1 g/L medium and was trypsinized after which the suspension cells (up to 1 × 10⁶) were collected via centrifugation at 250 × g for 5 min before discarding the medium. The cell pellet was resuspended in 300 µL of Binding Buffer and properly mixed using vortexing and pipetting followed by addition of 300 µL of 70% ethanol and vortex to ensure the homogeneity. The cell sample was transferred to a Spin Cartridge with a Collection Tube and centrifuged for 1 min at 12,000 × g. After discarding the flow–through, 700 µL of 96–100% ethanol was added, vortexed, and transferred to a fresh Spin Cartridge in two sequential steps. Centrifuge at 12,000 × g for 1 min after each addition. After removing the flow–through, the Spin Cartridge was placed in a Collection Tube. To eliminate remaining impurities, add 500 µL of Wash Buffer and centrifuge at 12,000 × g for 1 min. The washing process was repeated once. The Spin Cartridge was then placed in a Wash Tube and centrifuged at full speed for 2–3 min to remove any leftover ethanol. To elute miRNA, insert the Spin Cartridge in a clean 1.7 mL Recovery Tube and add 50–100 µL of sterile, RNase–free water to the membrane center. Incubate for 1 min before centrifugation at maximum speed for another min. The purified miRNA was collected in the Recovery Tube and used for downstream applications. The purity of the extracted miRNA was assessed using a NanoDrop spectrophotometer, which showed an OD260/280 ratio of 1.955, consistent with high-quality RNA.

**Fluorescence studies** **of IR–ssDNA–Pt–BDP + miRNA (from cells):** Fluorescence emission studies were conducted on IR–ssDNA–Pt–BDP + miRNA (from cells) where miRNA was annealed with IR–ssDNA–Pt–BDP in different ratio (IR–ssDNA–Pt–BDP: miRNA=1:1, 1:1.5, 1:2) before and after irradiation with NIR light. A FluoroMax–3 fluorescence spectrophotometer equipped with FluorEssence software was used to record the fluorescence spectra. Ascorbic acid (100 µM) and above–mentioned compounds (5 µM) solutions were made in PBS (pH~7) at room temperature in cuvettes in a dark environment. Emission spectra were recorded at 0 min (in the dark) as the initial fluorescence measurements followed by after irradiation at different intervals.

**Photoreduction of IR–dsDNA–Pt–BDP using PAGE gel–based assay:** To determine the photoreduction–induced uncaging of the IR–dsDNA–Pt–BDP construct, two identical samples (5 μM each) were generated. One sample was kept in the dark, while the other was exposed to 830-nm NIR light for 45 sec in the presence of 100 μM ascorbic acid as a reductant. A 16% PAGE gel was used to compare the two samples, with IR-dsDNA–S–S serving as a control. The gel was stained with SYBR Gold and scanned with a Typhoon scanner under both Alexa Fluor 488 and Cy-5 (650 nm) channels to analyze changes in band migration and fluorescence patterns. This confirmed photo-triggered BDP release from the DNA construct.

**Fluorescence-based stability assessment of Compound 2 in the dark:** The stability of Compound **2** was evaluated under various biologically relevant conditions using a Qubit™ 4 Fluorometer. Each sample was prepared at a final concentration of 5 μM in PBS buffer (pH 7.4). For stability testing, ascorbic acid (500 μM), glutathione (6 mM), solutions of complete DMEM medium (1 g/L glucose), and HeLa cell lysate were freshly prepared in PBS and mixed with the compounds. The mixtures were incubated at 37 °C in the dark, and aliquots were withdrawn at predetermined time points (0 h, 2 h, 4 h, 8 h, 12 h, and 24 h). Fluorescence intensity changes were recorded to monitor stability, using the free BDP reporter as a control.

**Cell imaging for assessing stability of Compound 2 in the dark *in vitro*:** HeLa cells were seeded in 35-mm sterile culture dishes (Celltreat Scientific Products) and cultured in DMEM supplemented with 10% FBS and 1% penicillin–streptomycin for 24 h under standard conditions (37 °C, 5% CO₂). Cells were then treated with **2** and the free BDP reporter (5 μM) separately. For time-dependent mitochondrial colocalization analysis, one set of dishes was incubated for 5 h, and a second set was incubated for 24 h. Following incubation, cells were stained with MitoTracker Red (0.5 μM) and Hoechst 33342 (2 μM) and incubated for an additional 30 min at 37 °C. Cells were subsequently washed twice with 1 mL of phosphate-buffered saline (PBS) and imaged live using a fluorescence microscope. Image analysis was carried out using ImageJ/Fiji. Colocalization between the green channel (BDP-based probes) and the red channel (MitoTracker) was assessed using the Coloc 2 plugin. The Pearson’s correlation coefficient (PCC) was calculated, and corresponding colocalization scatter plots were generated for each condition to evaluate spatial overlap between the probes and mitochondrial signal.

**Fluorescence-based assessment of IR–dsDNA–Pt–BDP stability and NIR-triggered functionality in cell culture medium:** The stability of IR–dsDNA–Pt–BDP was evaluated in cell culture medium using a Qubit™ 4 Fluorometer. IR800–dsDNA–Pt–BDP (5 μM) was incubated in complete DMEM medium supplemented with 10% (v/v) FBS and 1% (v/v) penicillin/streptomycin at 37 °C in the dark. At specified time points (0 h, 2 h, 4 h, 8 h, 12 h, and 24 h), aliquots were withdrawn and subjected to NIR light irradiation (830-nm LED, 30 seconds) under identical conditions to trigger BDP release. The fluorescence intensity corresponding to released BDP was quantified using the Qubit™ 4 Fluorometer.

**Cell imaging for validating functionality of the DNA-Pt scaffold *in vitro*:** HeLa cells were used to evaluate DNA-Pt construct’s functionality *in vitro*. IR-dsDNA–Pt–BDP, IR-ssDNA–Pt–BDP + Mutant-1, IR-ssDNA–Pt–BDP + Mutant-2, and IR-ssDNA–Pt–BDP (10 µM each) were added individually to sterile 35 mm culture dishes (Celltreat Scientific Products) and incubated at 4 °C for 3 h. Following pre-incubation, HeLa cells were seeded in DMEM supplemented with 10% (v/v) FBS and 1% (v/v) penicillin/streptomycin directly onto the DNA-containing dishes and incubated at 37 °C under 5% CO₂ for 7 h. The cells were subsequently washed twice with 1 mL PBS and imaged using a fluorescence microscope. Images were acquired at 0 sec (dark) and following NIR irradiation (830-nm LED) for 15 sec, 30 sec, and 45 sec, respectively.
